# Real‐Time Investigation of Interfacial Evolution in Electrochemical Energy Storage Systems via EQCM

**DOI:** 10.1002/advs.76503

**Published:** 2026-07-09

**Authors:** Zixuan Wang, Situo Cheng, Guang Feng

**Affiliations:** ^1^ School of Energy and Power Engineering Huazhong University of Science and Technology Wuhan P. R. China

**Keywords:** charge storage mechanism, EQCM, interface reaction and evolution, quartz resonator, solid–liquid interface

## Abstract

Understanding the structure and formation process of the electrode/electrolyte interface (EEI) is crucial for electrochemical energy storage research. The electrochemical quartz crystal microbalance (EQCM) has evolved into a powerful in situ characterization tool capable of resolving complex electrochemical interfacial processes. This review comprehensively summarizes recent progress in EQCM and its derivative techniques, including EQCM with dissipation monitoring and alternating current electrogravimetry (ac‐EQCM) for investigating the EEI. Special emphasis is placed on the role of EQCM in unraveling charge storage mechanisms, evaluating structural stability and failure modes, and monitoring interfacial reactions, including the formation and dynamics of solid‐electrolyte interphases. Additionally, recent breakthroughs in the joint use of EQCM with complementary techniques, such as spectroscopy and nuclear magnetic resonance, are discussed to highlight its unique advantages in multi‐dimensional analysis of interfacial mass, structural, and mechanical evolution. Finally, the future development of EQCM is explored, emphasizing its enduring significance in accelerating the development of next‐generation energy storage devices.

## Graphical abstract

Understanding the electrode/electrolyte interface (EEI) is crucial for supercapacitors and batteries. EQCM, by enabling real‐time monitoring of mass changes during charge–discharge cycles, has evolved into a vital method for analysis of EEI. It provides important insights into charge‐storage mechanisms, electrode structural evolution, ion‐transfer behavior, and interfacial deposition. Combined with other techniques, EQCM can even resolve more complex interfacial phenomena.

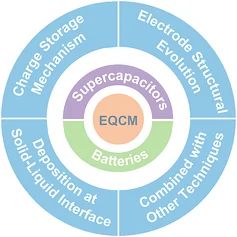

## 1 Introduction

While electrochemical energy storage device (EESD) technologies have achieved widespread commercialization, their current energy and power densities still struggle to meet the burgeoning performance demands of smart grids, electric vehicles, and next‐generation portable or wearable electronics [1, 2]. The charge storage capability of these devices is determined not only by the microscopic arrangement of electrolyte ions at the EEI, but more fundamentally by the intricate coupling between electron‐transfer processes and ion‐transport kinetics across the interface [3]. Therefore, despite the diversity of charge carriers and active materials across different energy storage systems, their fundamental physicochemical processes consistently converge on the electric‐field‐driven formation and dynamic evolution of the solid–liquid interface. It is thus evident that elucidating the interface structure and its formation mechanisms has become a central focus of electrochemical research [4]. However, the electrochemical interface exhibits complex coupling characteristics involving multiple concurrent dynamic processes, including ion adsorption/desorption, solvation/desolvation, charge transfer, and nucleation/dissolution, the superposition of which renders precise interfacial analysis highly challenging [5]. Accordingly, the development of in situ/operando characterization techniques that combine high interfacial sensitivity, superior spatiotemporal resolution, and the ability to closely approximate practical operating conditions is essential for real‐time tracking of interfacial evolution during electrochemical cycling [6, 7].

EQCM enables real‐time monitoring of interfacial reactions in electrochemical systems by correlating resonant‐frequency shifts with mass fluctuations at the electrode surface. Owing to its simple sample preparation, broad applicability, cost‐effectiveness, and non‐destructive nature, EQCM has become a versatile platform for interfacial characterization. Importantly, quantitative analysis of mass and viscoelasticity variations allows reconstruction of the structural evolution of both bulk and interfacial domains, thereby providing fundamental insights into charge storage mechanisms, ion/electron transfer kinetics, and the long‐term stability of EEI [8]. In recent years, with the deepening research into EESDs, such as lithium‐ion batteries [9, 10], aqueous zinc‐ion batteries [11, 12], and supercapacitors [13, 14], EQCM has been widely employed to probe EEI processes owing to its high sensitivity to nanogram‐level mass changes and film viscoelastic changes. In particular, it has played a pivotal role in elucidating charge storage mechanisms [15, 16, 17], uncovering ion transport behavior [18, 19], resolving electric double‐layer structures [20, 21, 22], and tracking the formation and dynamic evolution of interfaces [23, 24].

**FIGURE 1:**
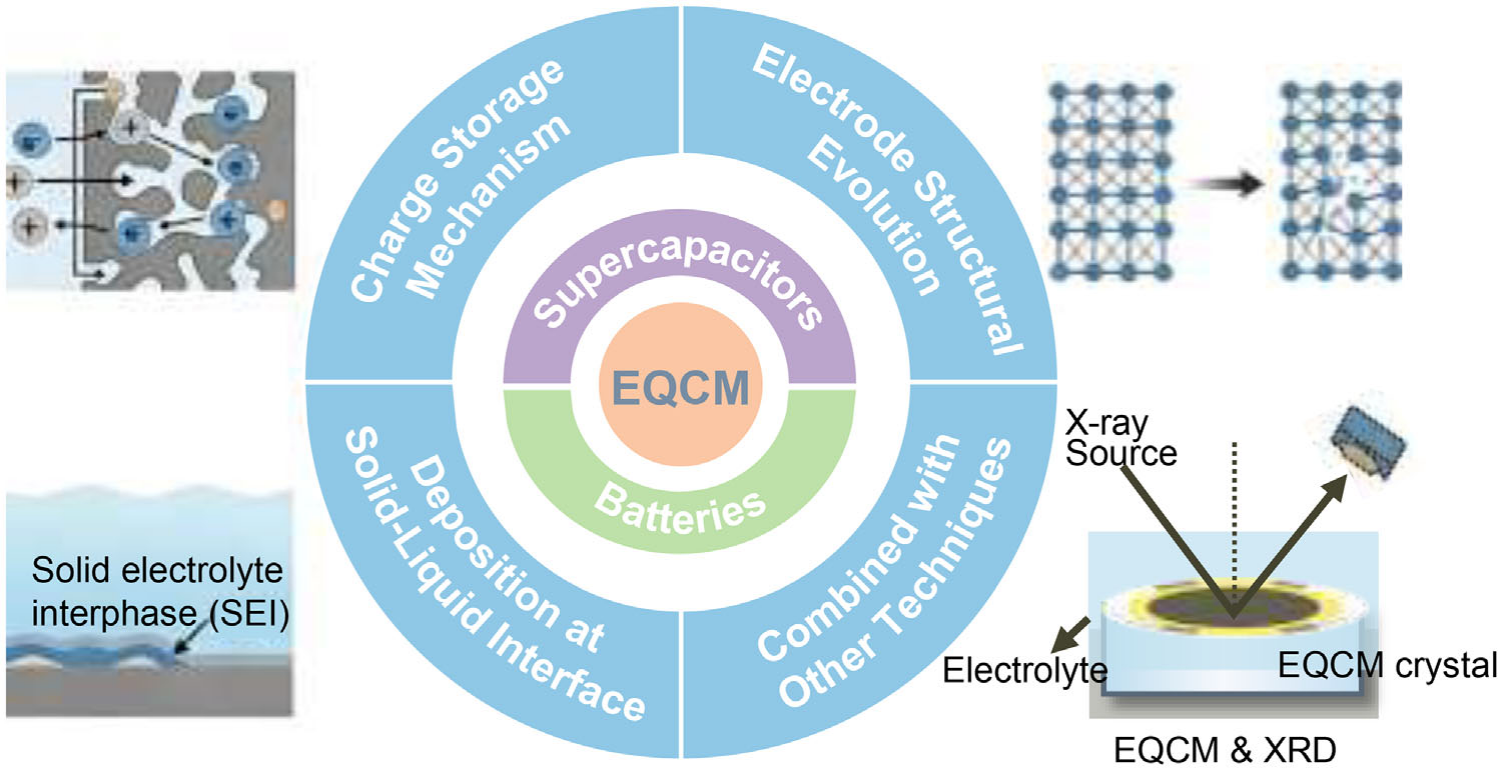
Schematic illustration of EQCM applications in energy storage.

In this review, we first briefly introduce the fundamental principles of EQCM, including classical gravimetric analysis, ac‐EQCM, and EQCM‐D, together with their respective data‐processing methodologies. Subsequently, we focus on specific applications of EQCM in supercapacitors and battery systems, as shown in Figure 1, covering in‐depth analyses of charge storage mechanisms, investigations of stress evolution in electrode structures, and dynamic monitoring of interfacial reaction processes. Furthermore, we summarize integration strategies that combine EQCM with various in situ characterization techniques (e.g., NMR, XRD, ellipsometry, XPS, OEMS/DEMS, spectroscopy, and electrochemical dilatometry) and theoretical methods, highlighting their synergistic value in resolving complex interfacial processes across multiple scales and dimensions. Finally, based on the current research landscape, we outline future development directions for EQCM in complex systems, multimodal integration, and intelligent data analytics, aiming to provide methodological guidance for the rational design and optimization of high‐performance energy storage devices.

## 2 Basic Principles of EQCM

### 2.1 Theory and Operational Principles

**FIGURE 2:**
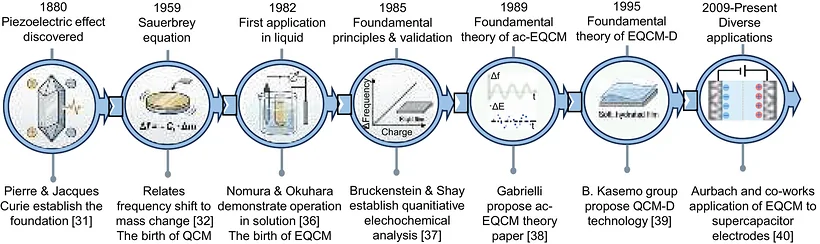
Scheme of historical milestones in EQCM technology development [31, 32, 36, 37, 38, 39, 40].

**FIGURE 3:**
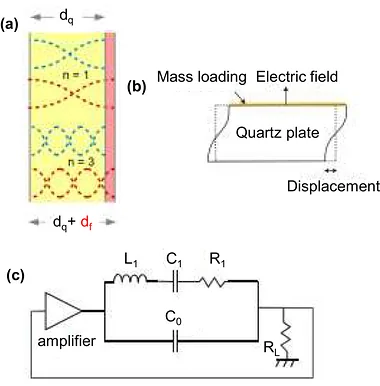
(a) Schematic diagram of transverse shear waves passing through thin films. Reproduced under the terms of the Creative Commons CC‐BY license [41]. Copyright 2021, D. Johannsmann et al. (b) Thickness shear mode of quartz crystal microbalance. Reproduced under the terms of the Creative Commons CC‐BY license [44]. Copyright 2021, S. Na Songkhla et al. (c) BVD equivalent circuit model, where *R*_1_ is motional resistance; *L*_1_ is motional inductance; *C*_1_ is motional capacitance, and *C*_0_ is parallel/static capacitance. Reproduced under the terms of the Creative Commons CC‐BY license [47]. Copyright 2023, S. Liao et al.

The development of the EQCM can be traced back to the 1880s, shown in Figure 2, when Jacques and Pierre Curie discovered the piezoelectric effect [25]. The fundamental operating principle of QCM relies on the inverse piezoelectric effect of single‐crystal quartz: when subjected to an alternating electric field, the crystal undergoes electromechanical coupling and generates stable acoustic oscillations [26]. As shown in Figure 3a, in a QCM system, acoustic waves propagate in a transverse shear mode; resonance occurs when half of the acoustic wavelength equals an odd multiple of the crystal thickness [27]. At present, AT‐cut quartz crystals are most widely employed in electrochemical energy storage studies, operating predominantly in the thickness shear mode.

In 1959, Günter Sauerbrey reported that an increase in mass rigidly attached to the surface of a piezoelectric quartz crystal under an alternating potential produces a proportional decrease in the resonance frequency, provided that the deposited layer is thin, uniform, mechanically rigid, and rigidly coupled to the crystal surface [28, 29, 30, 31]. This relationship laid the theoretical foundation for nanogram (ng)‐level mass sensing by QCM. The intrinsic ultrahigh sensitivity of the QCM arises from the exceptional frequency stability and resolution of the quartz oscillator, enabling the detection of mass variations potentially down to the sub‐monolayer level under suitable rigid‐film conditions [32].

Although QCM was initially applied mainly to mass monitoring in vapor‐phase deposition processes, Nomura and Okuhara demonstrated in 1982 that quartz crystals fully immersed in liquids can still sustain stable oscillations [33]. Subsequently, in 1985, Bruckenstein and Shay proposed a practical equivalent circuit model suitable for electrolyte environments, enabling synchronous control of electrode potential and quartz crystal oscillation [34, 35]. Since then, QCM has formally evolved into EQCM, establishing itself as a core in situ tool for disentangling complex electrochemical interfacial processes.

In the acoustic physics model, quartz crystals resonate in thickness shear mode as shown in Figure 3b. The characteristic wavelength of the fundamental acoustic wave λ_0_ equals twice the crystal thickness *d_q_* [41]:

(1) $$\lambda_{0}=2d_{q}$$

For an odd harmonic order *n*, the acoustic wavelength λ_*n*_ is inversely proportional to *n* [42]:

(2) $$\lambda_{n}=\lambda_{0}/n\;$$

Assuming an acoustic wave velocity ν and frequency *f*, according to ν  =  *f*λ_0_, the resonance frequency can be expressed as:

(3) $$f=\frac{n\nu }{2d_{q}}=\frac{n}{2d_{q}}\;\sqrt{\frac{\mu_{q}}{\rho_{q}}}$$

where ρ_*q*_ is the density of quartz (2.648 g/cm^3^) and μ_*q*_ is the shear modulus of quartz (2.947 × 10^11^ g/cm/s^2^).

From the standpoint of acoustic coupling, the shear acoustic waves generated in the thickness shear mode can penetrate rigid electrode coatings or thin films attached to the crystal surface. Under no‐slip conditions, the film oscillates synchronously with the quartz crystal. For a deposited film with thickness *d_f_*, the resulting mass loading induces a resonance frequency shift Δ*f* [43]:

(4) $$\frac{\Delta f}{f}=-\;\frac{d_{f}}{d_{q}}=-\frac{\Delta m}{m_{q}}$$

This relation provides a physical explanation for the decrease in resonance frequency induced by rigid thin‐film deposition [44]. However, when the crystal is immersed in a liquid environment or loaded with a non‐rigid, viscoelastic film, the system behavior becomes considerably more complex. In such cases, significant energy dissipation occurs within the film, manifested as a broadening of the resonance peak (denoted by *W* or Γ). In addition to the dissipation factor (∆*D* or *D*), the motional resistance (*R*_m_) derived from the equivalent Butterworth–Van Dyke (BVD) circuit model is also commonly used to characterize energy dissipation behavior in QCM‐based measurements, as shown in Figure 3c [45, 46, 47]. Variations in *R*_m_ are closely associated with viscous loading and interfacial viscoelastic effects on the crystal surface. The coupled responses of resonance frequency shifts (Δ*f*) and energy dissipation changes (Δ*D*, ΔΓ, or *R*_m_) jointly encode rich information on the mechanical properties of electrode materials and the evolution of interfacial structures, forming the theoretical basis for EQCM‐D analysis.

### 2.2 EQCM Testing Method

#### 2.2.1 Traditional Gravimetric Measurement Method

During EQCM measurements, the frequency‐derived mass information is reliable only when the electrode coating behaves as a rigid load, that is, when the dissipation factor D remains unchanged during electrochemical operation. Under such conditions, two essential criteria must be satisfied: (1) the electrode film must be firmly attached to the quartz crystal surface and remain rigid throughout both open‐circuit and polarized states; (2) the porosity or volume of the electrode should not vary significantly with potential, as potential‐dependent structural changes would alter the interfacial hydrodynamic conditions and induce variations in the dissipation factor within the boundary layer [48]. When these conditions are fulfilled, the Δ*f* exhibits a direct proportionality to the evolution of interfacial mass. Under such conditions, the Sauerbrey equation can be rigorously applied to perform a quantitative conversion from frequency response to the mass fluctuations occurring during electrochemical processes.

(5) $$\Delta f=-\frac{2f_{0}^{2}}{A\sqrt{\rho_{q}\mu_{q}}}\Delta m$$

where Δ*f* is the resonance frequency shift, *f*_0_ is the fundamental resonance frequency of the quartz crystal, *A* is the effective surface area, ρ_*q*_ is the density of quartz (2.648 g/cm^3^), μ_*q*_ is the shear modulus of quartz (2.947 × 10^11^ g/cm/s^2^), and Δ*m* is the mass change of the electrode film.

The mass sensitivity of EQCM is closely related to the fundamental frequency of the crystal. According to Equation (5):

(6) $$\Delta f=-C_{f}\Delta m$$

where the mass sensitivity constant *C_f_* is given by:

(7) $$C_{f}=\frac{2f_{0}^{2}}{A\sqrt{\rho_{q}\mu_{q}}}\;$$

Therefore, the sensitivity is proportional to the square of the frequency ($C_{f}\;\propto \;f_{0}^{2}$). For AT‐cut quartz crystals, under typical operating conditions, the mass change corresponding to a unit frequency shift is given in Table 1.

**TABLE 1:** Theoretical gravimetric sensitivity of EQCM at various resonant frequencies.

Fundamental frequency (*f*_0_)	Mass change per 1 Hz frequency shift
5 MHz	17.7 ng/cm
6 MHz	12.3 ng/cm
9 MHz	5.5 ng/cm
10 MHz	4.4 ng/cm

By correlating the Δ*m* with the corresponding charge consumed (Δ*Q*) according to Faraday's law, a critical parameter in EQCM analysis, mass accumulated per mole of electrons (MPE) can be derived.

(8) MPE=nFΔm/ΔQ

where *n* denotes the number of electrons transferred in the reaction, and *F* is the Faraday constant (96485 C/mol). Comparison of the experimentally obtained MPE values with the molar masses of possible ionic or molecular species enables identification of the dominant charge carriers involved in the electrochemical process.

Despite its exceptional versatility, EQCM has an inherent limitation: it lacks intrinsic chemical selectivity. In complex multicomponent systems, the measured mass change generally reflects the net contribution of multiple interfacial processes, making it difficult to directly assign the observed response to specific ions, solvent molecules, or reaction intermediates without complementary characterization [49]. To address this limitation, Lin et al. proposed a deconvolution approach based on EQCM data, enabling quantitative separation of the real‐time fluxes and corresponding ionic currents of different species [50].

Assuming that *i* species (*i* is an integer) participate in an electrochemical process, the mass change of the electrode is the sum of the masses of all participating species. According to the laws of mass conservation and charge conservation, the total mass change Δ*m* and charge change Δ*Q* at a given time *t* can be expressed as:

(9) $$\Delta m=N_{t_{1}}\;M_{t_{1}}+N_{t_{2}}M_{t_{2}}+\cdot \cdot \cdot N_{t_{i}}M_{t_{i}}$$

(10) $$\Delta Q=-\left(N_{t_{1}}x_{t_{1}}+N_{t_{2}}x_{t_{2}}+\cdots \cdots \cdots N_{t_{i}}x_{t_{i}}\right)F$$

where *N* and *M* denote the molar amount and molar mass of each species, respectively, and *x* represents the valence state. The participating species can be anions, cations, or solvent molecules; therefore, *x* can be positive, negative, or zero. Since the charge accumulated on the electrode is opposite to the ionic charge, a negative sign is introduced in Equation (10).

Δ*m* and Δ*Q* can be obtained by EQCM, and the corresponding expressions constitute a set of multivariate linear equations. When the number of participating species is less than or equal to two, the equations have a unique solution, allowing the time‐dependent quantities of individual species to be determined. Since EQCM and electrochemical data are recorded at discrete time intervals. If k data points are collected up to time t, the mass and charge changes at all time steps can be expressed as:

(11) $$A=\left[\begin{array}{cc} \Delta m_{t_{1}}\;\Delta m_{t_{2}}\;\text{…} & \Delta m_{t_{k}}\\ \Delta Q_{t_{1}}\;\Delta Q_{t_{2}}\;\text{…} & \Delta Q_{t_{k}} \end{array}\right]=BX$$

where

(12) $$B=\left[\begin{array}{cc} M_{1} & M_{2}\\ -x_{1}F & -x_{2}F \end{array}\right]\;$$

(13) $$X=\left[\begin{array}{cc} N_{1t_{1}}\;N_{1t_{2}\;}\text{…} & N_{1t_{k}}\\ N_{2t_{2}}\;N_{2t_{2}\;}\text{…} & N_{2t_{k}} \end{array}\right]\;$$

here, the matrix *A* represents the data on the changes in mass (Δ*m_t_*) and charge (Δ*Q_t_*) at all measurement time points obtained from EQCM and electrochemical testing. Matrix *B* represents the molecular weight (*M_i_*) and valence state (*x_i_*) of the participating substances. Matrix *X* represents the population size of the participating substance (*N_it_*). Furthermore, species flux (*dN*/*dt*) can be given by the derivative of quantitative changes over time.

To obtain species fluxes, the discrete data in *X* must be fitted with continuous and differentiable functions. Polynomial fitting (e.g., a fifth‐order polynomial *F*(*t*)) is commonly employed, and the instantaneous flux *J* (mol/s) of species *i* can be calculated as:

(14) $$J\;\left(t_{i}\right)=\frac{dF{\left(t\right)}_{i}}{dt}\;$$

During cyclic voltammetry, the measured current arises from the influx and efflux of charge carriers at the electrode. The total current can therefore be decomposed into contributions from individual ionic species, with the ionic current of species *i* given by:

(15) $$I_{ioni}=\frac{dQ}{dt}=\frac{dNF\left(-x_{i}\right)}{dt}=J{\left(t\right)}_{i}F\left(-x_{i}\right)$$

After obtaining the real‐time ionic currents of different species, ionic current cyclic voltammetry curves can be constructed, enabling a more intuitive comparison of the real‐time current contributions from different ions.

As can be clearly seen from the derivation of the above equations, the accuracy of the calculated results largely depends on the precision of the mass and charge changes measured by EQCM. Therefore, repeated measurements on the target electrode are required to ensure the reliability of the results.

#### 2.2.2 Ac‐Electrogravimetry

**FIGURE 4:**
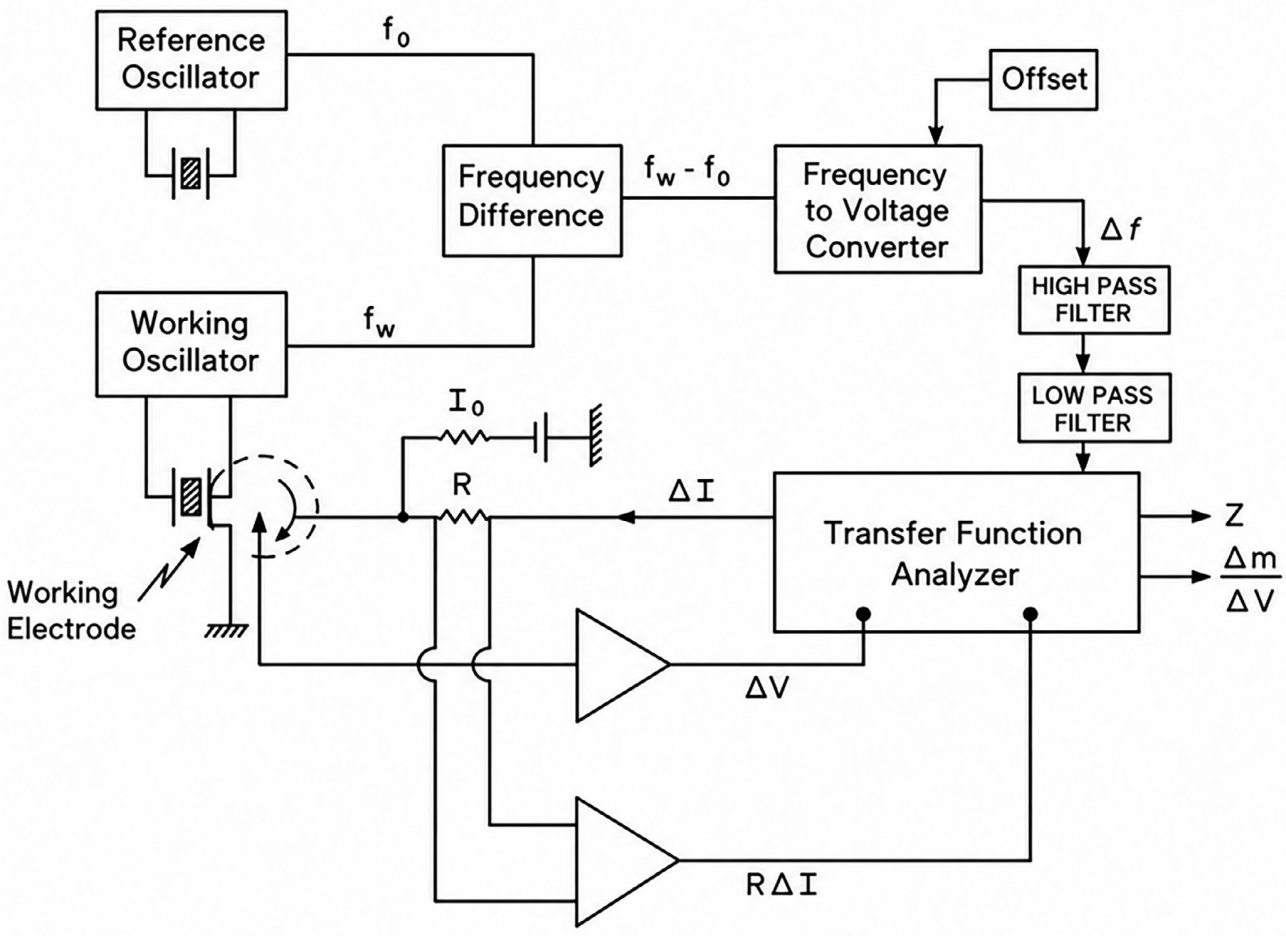
Schematic illustration of the ac‐EQCM principle. Reproduced with permission [38]. Copyright 1989, Elsevier Ltd.

Compared with conventional gravimetric EQCM, *ac*‐electrogravimetry (ac‐EQCM) is an alternative and complementary characterization technique. By combining EQCM with electrochemical impedance spectroscopy (EIS), it enables exploring complex electrochemical mechanisms [51, 52, 53]. The measurement is performed by applying a small sinusoidal potential perturbation at a fixed potential, allowing the frequency‐dependent mass and charge variations to be obtained. The measurement principle of ac‐EQCM is schematically illustrated in Figure 4 [38]. These data are then used to generate the classical EIS transfer function (TF) Δ*E*/Δ*I*(ω), which is typically expressed in the form of the charge‐potential transfer function Δ*q*/Δ*E*(ω), as well as the mass‐potential transfer function Δ*m*/Δ*E*(ω) [54]. A low‐amplitude sinusoidal potential perturbation (Δ*E*) induces sinusoidal concentration fluctuations (Δ*C_i_*) of each species within the electrode/electrolyte system, thereby enabling the analysis of frequency‐dependent interfacial transfer dynamics and species‐specific contributions to the overall electrochemical response. The theoretical framework of this approach was systematically established by Gabrielli et al. [55, 56], and can be expressed as follows:

(16) $$\frac{\Delta C_{i}}{\Delta E}\;\left(\omega \right)=\frac{-G_{i}}{j\omega d_{f}+K_{i}}\;$$

(17) $$\frac{\Delta E}{\Delta I}\;\left(\omega \right)={\left(j\omega d_{f}F\sum_{i}\frac{G_{i}}{j\omega d_{f}+K_{i}}\right)}^{-1}\;\left(i\;\mathrm{is}\;\mathrm{ions}\right)$$

(18) $$\frac{\Delta q}{\Delta E}\;\left(\omega \right)=Fd_{f}\sum_{i}\frac{G_{i}}{j\omega d_{f}+K_{i}}\;\left(i\;\mathrm{is}\;\mathrm{ions}\right)$$

(19) $$\frac{\Delta m}{\Delta E}\;\left(\omega \right)=-d_{f}\sum_{i}M_{i}\frac{G_{i}}{j\omega d_{f}+K_{i}}\;\left(i\;\mathrm{is}\;\mathrm{ions}\;\mathrm{and}\;\mathrm{solvent}\right)$$

where Δ*C_i_* represents the concentration variation of species, ω  =  2π*f*, *d_f_* is the film thickness, *K_i_* and *G_i_* correspond to the partial derivatives of the interfacial flux *J_i_* of species *i* with respect to concentration and potential, respectively, defined as *K_i_*  =  (∂*J_i_*/∂*C_i_*)_*E*_, $G_{i}={\left(\partial J_{i}/\partial E\right)}_{C_{i}}$. Here, *K_i_* reflects the intrinsic kinetic rate of interfacial transfer. Whereas *G_i_* is the inverse of the transfer resistance. *M_i_* denotes the molar mass of the corresponding species.

Ac‐EQCM analysis can provide the following unique information: (i) the kinetics of mass transport and interfacial transfer at the EEI; (ii) identification of species based on their molar mass, enabling clear distinction among anions, cations, solvated ions (and their solvation numbers), and free solvent molecules; and (iii) the relative concentration changes of these species within the electrode material. The dynamic characterization of interfaces and the precise identification of different species involved in (electro)chemical processes represent key advantages of advanced ac‐EQCM analysis [57].

**FIGURE 5:**
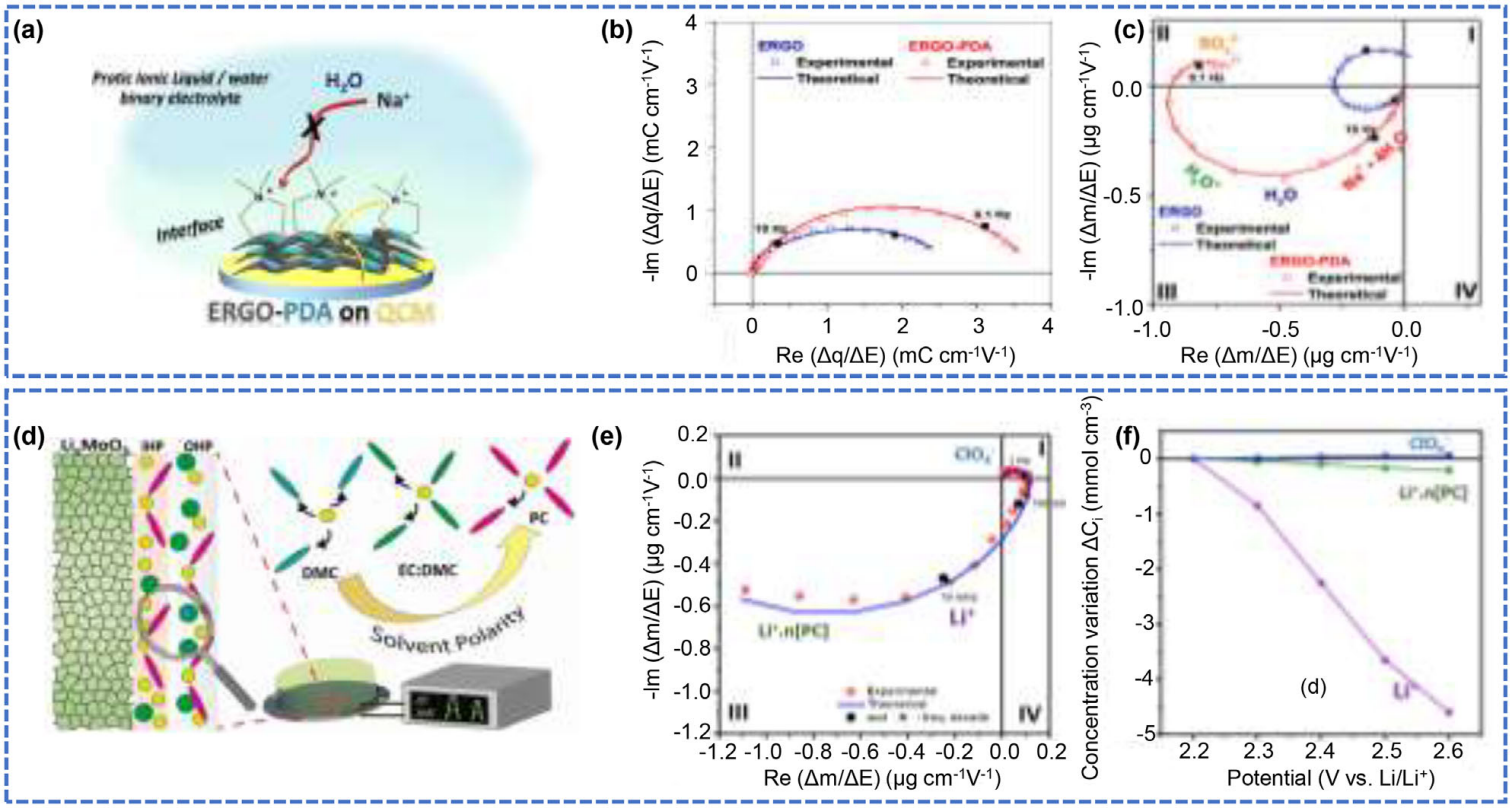
(a) Schematic illustration of the ERGO‐PDA modified QCM at the electrode/electrolyte interface; (b) Experimental and theoretical *ac*‐electrogravimetric data of ERGO and ERGO‐PDA at 0 V versus SCE: potential charge transfer function Δ*q*/Δ*E*(ω) and (c) mass potential transfer function Δ*m*/Δ*E*(ω). Reproduced with permission [61]. Copyright 2022, American Chemical Society. (d) Schematic illustration of the Li*_x_* MoO_3_/electrolyte interface, showing the electric double layer (EDL) structure (IHP and OHP) and the effect of solvent polarity. (e) The experimentally measured Δ*m*/Δ*E*(ω) transfer function (TF) is fitted by the transfer contributions of three substances (Li^+^, Li^+^·*n*[PC], where *n *= 1, and ClO_4_^−^) at the EEI interface; (f) The concentration changes of each substance are estimated according to Equations (16) and (19). Reproduced with permission [60]. Copyright 2022, American Chemical Society.

Since the development of ac‐EQCM by Gabrielli and Perrot, this technique has been extensively advanced and applied to energy‐storage interfaces, particularly through the contributions of Perrot, Sel, and co‐workers [39, 58]. Compared with conventional EQCM, which mainly records the net mass variation during electrochemical processes, ac‐EQCM provides dynamic transfer functions that can separate coupled charge‐compensation processes when the involved species exhibit different kinetic time constants. If the time constants of the different charged species are distinct, they appear as separate semicircles. If they are similar, depressed semicircles are obtained. Fitting these experimental transfer functions with Equations ((15), (16), (17)) can then decouple the contributions from ion transfer, solvent participation, and interfacial dynamics. This capability has been demonstrated in several energy‐storage systems, including vertically oriented graphene oxide nanosheets in non‐aqueous electrolytes [54], SWCNT‐based capacitive electrodes in aqueous electrolytes [58], pseudocapacitive MnO_2_ electrodes [59], and lithium‐ion battery interfaces based on model Li*_X_* MoO_3_ materials [60]. These studies established ac‐EQCM as a powerful approach for resolving charge‐compensation mechanisms, solvation/desolvation processes, and interfacial mass‐transfer dynamics in batteries and supercapacitors. Specifically, ac‐EQCM was utilized to decipher the intricate interfacial processes of graphene and graphene‐polydopamine (ERGO‐PDA) composite electrodes in various electrolytes. To better illustrate the investigated process, Figure 5a schematically shows the ERGO‐PDA electrode supported on a QCM substrate and the electrolyte species involved at the electrode/electrolyte interface. Figure 5b,c present the experimental and theoretical charge‐potential Δ*q*/Δ*E*(ω) and mass‐potential Δ*m*/Δ*E*(ω) transfer functions at 0 V versus SCE. Excellent agreement between experimental data and theoretical fitting was observed in both spectral shape and characteristic frequency. Through frequency‐domain analysis, ac‐EQCM enabled the separation and quantification of the contributions from different interfacial species, thereby providing detailed insight into species‐specific interfacial transfer kinetics and charge‐compensation processes, as schematically illustrated in Figure 5c. The analysis revealed the participation of three charged species, Na^+^·4H_2_O, H_3_O^+^, and SO_4_^2−^ in the adsorption process, each exhibiting distinct frequency‐dependent interfacial transfer kinetics. Specifically, hydrated Na^+^ dominated fast interfacial exchange in the high‐frequency region, H_3_O^+^ contributed predominantly at intermediate frequencies. While SO_4_^2−^ governed the low‐frequency response in the fourth quadrant, corresponding to slower kinetics. Moreover, the kinetic constants of free H_2_O molecules were found to be comparable to those of hydrated Na^+^, indicating that free water molecules are co‐transported into the interface via an electro‐dragging effect. Importantly, separating the individual contributions of different species can help explain discrepancies between the mass variation estimated from Faraday's law and the actual mass response measured by EQCM. These results demonstrate the unique capability of ac‐EQCM to deconvolute classical EQCM responses into species‐specific contributions and quantitatively resolve complex multi‐species interfacial dynamics in the frequency domain [61]. The same methodology was further applied to elucidate the electrical double‐layer (EDL) structure and interfacial kinetics of Li_x_MoO_3_ electrodes. As schematically illustrated in Figure 5d, the Li_x_MoO_3_/electrolyte interface involves a complex EDL structure, where bare Li^+^, solvated Li^+^‐solvent complexes, and anions can participate in charge compensation at different interfacial locations and with different kinetic behaviors. As shown in Figure 5e,f, three charge‐compensating species were identified: bare Li^+^, solvated Li^+^‐solvent complexes, and anions (ClO_4_^−^ or TFSI^−^). The effects of solvent polarity on the ion solvation state and the EDL structure were analyzed. In highly polar solvents, Li^+^ tends to exist in a solvated form, whereas in low‐polarity solvents, ion‐pair formation is favored, which suppresses anion migration. A “quasi‐specific adsorption” mechanism was further proposed to explain the reversible adsorption behavior of anions at the interface. This study demonstrates the strong capability of ac‐EQCM in revealing complex interfacial charge compensation mechanisms [60].

#### 2.2.3 Non‐Gravimetric Method

The classical Sauerbrey equation assumes an ideal, rigid, and thin film, in which the electrode loading oscillates synchronously with the quartz crystal. However, under practical operating conditions of energy storage devices, the presence of soft interfacial layers, dynamic variations in electrolyte properties, and the interference of longitudinal wave effects cause the electrode system to exhibit pronounced viscoelastic behavior. As a result, the Sauerbrey model is no longer applicable. To quantify the effects of non‐rigid interfaces, Kanazawa and Gordon [37, 62] proposed a frequency response model in 1985 that accounts for the viscosity and density of the solution close to the interface. From the perspective of fluid dynamics, the differences between liquids lie in their density (ρ) and dynamic viscosity (η), and this model shows that the frequency shift (Δ*f_L_*) and motional resistance changes (Δ*R*) caused by the liquid load are given by:

(20) $$\Delta \;f_{L}=-{f_{0}}^{3/2}{\left[\rho_{L}\eta_{L}/\left(\pi \mu_{q}\rho_{q}\right)\right]}^{1/2}$$

(21) $$\begin{array}{c} \Delta R={\left[2\pi f_{0}\Delta \left(\rho_{L}\eta_{L}\right)\right]}^{\frac{1}{2}}A/k^{2}=-\left[\pi {\left(2\mu_{q}\rho_{q}\right)}^{\frac{1}{2}}A/\left(kf_{0}\right)\right]\Delta f_{L} \end{array}$$

where Δ*f_L_* is the frequency shift caused by the liquid, Δ*R* is the change in resonant resistance, ρ_*L*_ and η_*L*_ represent the density and viscosity of the liquid coupled to the quartz crystal surface, and *k* is the electromechanical factor. The physical essence of this equation is that shear waves dissipate energy in the viscous fluid. Currently, studies on viscoelastic thin films have mainly led to two analytical paradigms.

One approach, proposed by Dmitrij in 2002, enables impedance analysis by measuring the resonance peak width (*W* or Γ) of a damped crystal and first clarified the influence of specific electrode surface roughness features on QCM experiments [63]. It was initially termed electrochemical quartz crystal admittance (EQCA) analysis. In this framework, the resonance response is commonly characterized by the full width at half maximum, which is equivalent to the half‐width at half maximum or the dissipation‐related peak broadening parameter *W* (or *Γ*). EQCA analyzes the admittance spectrum in the vicinity of the resonance frequency, providing access to both frequency and dissipation‐related information. EQCA analyzes the admittance spectrum around resonance. In QCM‐based measurements in liquid, including EQCA, the oscillatory shear motion generates a shear wave that decays exponentially into the adjacent liquid. The penetration depth (δ) of these waves in the fluid can be expressed as follows [44, 64]:

(22) $$\delta ={\left(\frac{\eta_{L}}{\pi nf_{0}\rho }\right)}^{\frac{1}{2}}$$

From this equation, it can be seen that δ depends on the viscosity‐to‐density ratio (η/ρ) of the fluid and the harmonic order (*n*). According to the Kanazawa equation, for an ideal flat electrode, Δ*f* and Δ*W* scale linearly with the penetration depth δ. The changes in Δ*f*/*n* and Δ*W*/*n* are proportional to $\sqrt{\rho_{L}\eta_{L}}$ [65, 66],

(23) $$\Delta f=-\delta \frac{n\rho_{L}f_{0}^{2}}{\sqrt{\mu_{q}\rho_{q}}}$$

(24) $$\Delta W=\delta \frac{2n\rho_{L}f_{0}^{2}}{\sqrt{\mu_{q}\rho_{q}}}$$

**FIGURE 6:**
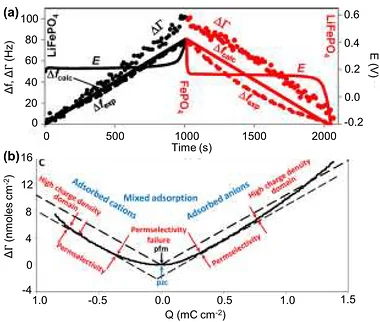
(a) Constant current charge test of Li*_x_* FePO_4_ in pure 0.5 m Li_2_SO_4_ solution and the corresponding EQCA components. The solid lines, triple solid lines, and filled circles correspond to Δ*f* mass, Δ*f_exp_*, and ΔΓ, respectively. Reproduced with permission [69]. Copyright 2013, American Chemical Society. (b) Δ*Γ* versus *Q* curve (solid line) for TiC‐CDC carbon electrode in 0.025 m KCl solution. The dashed lines represent the Faradaic line for K^+^ and Cl^−^ ion adsorption. Reproduced with permission [70]. Copyright 2013, American Chemical Society.

When the electrode interface deviates from the ideal plane assumption (e.g., due to structural reconstruction, pore evolution, or increased roughness), the Δ*f*–δ and Δ*W*–δ curves will deviate from a linear trajectory. Such analysis of the deviations in the Δ𝑓–𝛿 and Δ𝑊–𝛿 relationships requires multiharmonic EQCM‐D measurements, since reconstruction of the electrode microstructure and evolution of the mechanical modulus cannot be reliably achieved using only a single harmonic or the fundamental frequency [67, 68]. In energy storage systems, this method can directly measure the frequency shifts (Δ*f_exp_*) and resonance peak width changes (Δ*W* or ΔΓ) driven by potential during charge/discharge processes. The resulting responses can be used to evaluate the mechanical properties of the interfacial layer, provided that appropriate viscoelastic models, such as the Voigt‐based viscoelastic model, are applied. By correlating the coupled frequency and dissipation variations with parameters including viscosity, elasticity, and interfacial rigidity, the mechanical behavior within the interface can be further revealed. Levi et al. employed EQCA analysis to monitor ion (de)intercalation in LiFePO_4_ electrodes (Figure 6a), where concurrent dissipation responses provided the first evidence of ion migration‐induced non‐uniform deformation of the electrode [69]. In addition, this technique, combined with the electric double layer model and capacitive deionization theory (Figure 6b), successfully explains the confined adsorption behavior of ions in mesoporous and microporous carbon electrodes [70]. It provides a critical mechanical characterization dimension for analyzing complex electrode failure mechanisms.

Another method is the QCM with dissipation monitoring (QCM‐D) technique proposed by B. Kasemo's group at Chalmers University of Technology in Sweden in 1995 [39, 42]. This technique is commonly implemented using a ring‐down (decay) approach, in which the oscillation of the quartz crystal is excited and then allowed to freely decay after the driving AC voltage is interrupted [39]. Analysis of the decay process enables real‐time determination of both the resonance frequency and energy dissipation, providing insight into energy loss from the oscillating system into the surrounding medium [71]. At present, commercial QCM‐D systems (e.g., Biolin Scientific, Sweden) enable simultaneous multi‐harmonic measurements of frequency and dissipation (*D*), which represents a key advantage over single‐frequency QCM techniques by improving the reliability of interfacial viscoelastic characterization. This capability has led to its widespread application in electrochemical energy storage, polymer science, biochemistry, and sensor‐related fields [72, 73, 74, 75, 76].

The dissipation factor (D) is defined as the ratio of the energy dissipated (*E_dissipated_*) to the stored energy (*E_stored_*) in one period, multiplied by 2π, which is the reciprocal of the quality factor (Q factor) [77]:

(25) $$D=\frac{1}{Q}=\frac{E_{dissipated}}{2\pi E_{stored}}\;$$

The dissipation shift (Δ*D*) and the relationships between the full width of the resonance peak (Δ*W*) and the frequency shift (Δ*f*) are given by:

(26) $$\Delta D=\left(\frac{\Delta W}{n}\right)/\left(\frac{\Delta f}{n}\right)$$

This directly reflects the viscoelasticity of the electrode coating and crystal interface [78]. The uniqueness of QCM‐D lies in that it not only tracks mass changes of the electrode through Δ*f*, but also provides quantitative information on the mechanical flexibility and structural rigidity of surface films and internal structures of composite materials via dynamic monitoring of the Δ*D*  [79, 80]. For rigid layers, the Δ*W*/*n* values for different harmonics are consistent, that is, Δ*D*  =  0. For viscoelastic coatings, the frequency and dissipation factor changes depend on the harmonic order *n*. In this case, ΔD≠0, and mass changes cannot be described by the Sauerbrey equation but must be fitted using an appropriate viscoelastic model [81, 82, 83]. The key roles of ∆*D* in energy storage include: (1) Rapid identification of rigid and viscoelastic materials; (2) Tracking the structural changes of polymer binders; (3) Monitoring the structural stability of electrochemically formed phases within the electrode, such as ion‐intercalated phases or deposited species generated during electrochemical cycling; (4) Evaluating the mechanical stability of the interface film. The change in ∆*D* is a critical indicator for assessing the rigidity or viscoelasticity of electrode surface deposits (such as solid electrolyte interphase layer; SEI films).

## 3 Application of EQCM in Supercapacitors and Batteries

During the charge–discharge process, both double‐layer capacitance and Faradaic electrochemical reactions (electron‐induced redox processes) significantly influence the final structure and chemical composition of the interface. These factors subsequently dictate the structural integrity of the electrode, the reversibility of long‐term cycling [84], and the overall safety of the device [85]. The EQCM technique, through real‐time and highly sensitive monitoring of Δ*m* and Δ*Q* during electrochemical cycling, enables the quantitative analysis of charge storage mechanisms at the molecular level. It reveals key dynamic processes such as ion adsorption/desorption, intercalation/deintercalation, solvation/desolvation, structural changes of the electrode, phase transitions, and interfacial film (SEI/CEI) formation. Its unique advantage lies in directly correlating macroscopic electrochemical signals (current and voltage) with microscopic mass transport, providing direct experimental evidence for understanding the performance‐limiting factors of energy storage devices, such as supercapacitors and lithium/sodium/zinc‐ion batteries. Additionally, complementary use of EQCM with various in situ characterization techniques (such as NMR, EIS, XRD, and spectroscopy) and computational methods (e.g., molecular dynamics simulations) enables multidimensional and multiscale analysis of complex interfacial processes. This facilitates the development of high‐performance electrolyte designs, electrode structural optimization, and interfacial engineering strategies.

### 3.1 Charge Storage Mechanism

Identification of charge carriers and elucidation of ion coordination dynamics at the EEI are central to understanding charge storage mechanisms in EESDs. By simultaneously monitoring electrode Δ*m* and Δ*Q*, and by calculating the mass accumulated per mole of electrons (MPE), EQCM provides direct insights into the nature of ionic species (e.g., H^+^, Zn^2+^, Na^+^) and their solvation states involved during charging and discharging processes. This capability may help attribute the contributions of different species to specific electrochemical processes, such as electric double‐layer capacitance, pseudocapacitive behavior, and battery‐type intercalation, thereby providing insight into complex multistep and multi‐ion cooperative storage mechanisms.

#### 3.1.1 Ion Adsorption and Desorption

**FIGURE 7:**
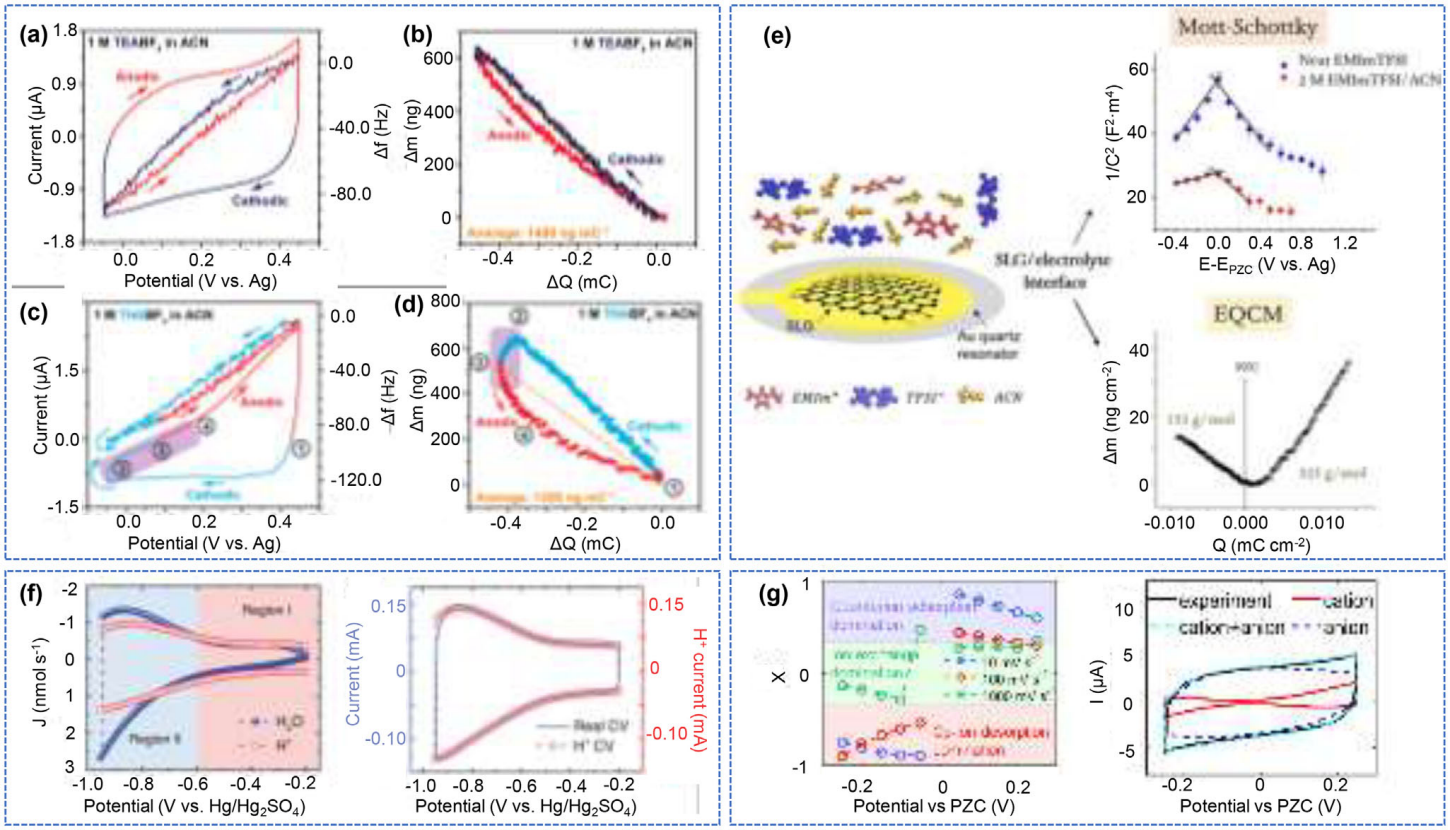
(a) CV and EQCM frequency response of Cu_3_(HHTP)_2_ in 1 m TEABF_4_ acetonitrile electrolyte. (b) The relationship between electrode Δ*m* and accumulated charge (Δ*Q*) calculated from the frequency response shown in (a). Reproduced under the terms of the Creative Commons CC‐BY 4.0 license [86]. Copyright 2024, American Chemical Society. (c) Cu_3_(HHTP)_2_ in 1 m THABF_4_ acetonitrile electrolyte. (d) The relationship between Δ*m* and Δ*Q* calculated from the frequency response shown in (c) [86]. (e) Mott‐Schottky analysis of SLG supported on Au‐coated quartz in neat EMIMTFSI and 2 m EMIMTFSI/ACN electrolytes. The relationship between electrode mass change and charge during the polarization of SLG on an Au electrode in 2 m EMIMTFSI/ACN electrolyte (scan rate 10 mV/s). Reproduced under the terms of the Creative Commons CC‐BY‐NC‐ND license [87]. Copyright 2021, John Wiley and Sons. (f) J–CV curve of H_2_O and H^+^, and the fitted CV curve of H^+^ compared with the experimental CV. Reproduced with permission [50]. Copyright 2022, John Wiley and Sons. (g) Variation of charging mechanism parameter *X* with potential at different scan rates, with anion and cation fitted current curves at a scan rate of 10 mV/s and the experimental CV. Reproduced with permission [91]. Copyright 2025, American Chemical Society.

EQCM serves as a powerful tool for quantitative, molecular‐level analysis of ion adsorption and desorption processes at electrode materials, allowing clear differentiation between anionic and cationic contributions to charge compensation. Forse and co‐workers employed EQCM to investigate the influence of electrolyte cation size on the charging mechanisms of layered metal–organic framework (MOF) supercapacitors. As shown in Figure 7a–d, smaller tetraethylammonium (TEA^+^) cations lead to higher capacitance and more symmetric charging behavior, whereas larger tetrahexylammonium (THA^+^) cations induce pore saturation and pronounced charging asymmetry. The mass responses provide direct evidence for the relationship between ion size and electrochemical performance, offering quantitative guidance for electrolyte–electrode matching in MOF‐based systems [86]. Tsai et al. used EQCM to investigate the relationship between ion size and charge‐storage behavior in nanoporous carbon electrodes. They found that 0.65 nm pores induced partial desolvation of EMIM^+^, whereas 1 nm pores allowed more freely solvated ion transport. EQCM further revealed the influence of pore structure on ion adsorption behavior, providing quantitative guidance for electrolyte–electrode matching [20]. Sel et al. used ac‐EQCM to study ion‐transfer behavior in vertically oriented graphene nanosheets in organic electrolytes. They quantitatively distinguished the dynamics of large TBA^+^ cations and small BF_4_^−^ anions, and found that BF_4_^−^ showed faster transfer kinetics and lower transfer resistance, dominating the charge‐storage process. This work demonstrates the important role of EQCM in understanding ion‐electrode matching and optimizing electrochemical performance [58]. Simon and co‐workers combined EIS with EQCM to examine the electrochemical behavior of single‐layer graphene (SLG) electrodes in neat ionic liquids and their organic mixtures (Figure 7e). The results indicate that the introduction of organic solvents markedly increases the carrier density at the SLG/ionic liquid interface. Unlike the ion‐exchange‐dominated behavior typically observed in amorphous porous carbons, SLG exhibits counter‐ion adsorption as the dominant charge storage mode under both positive and negative polarization, attributable to its sp^2^‐hybridized carbon framework [87]. Rui et al. fabricated an aqueous asymmetric pseudocapacitor based on conjugated polymer electrolytes and Ti_3_C_2_T*_x_* MXene, and employed EQCM to elucidate its charge storage mechanism. The results reveal a co‐ion desorption process: during charging, negatively charged sulfonate side groups compensate the increasing positive charge on the polymer backbone while expelling cations; during discharging, cations migrate back into the polymer electrode to maintain electroneutrality [88]. By integrating the surface functional groups of carbon materials with the electrolyte pH, Taberna and co‐workers demonstrated that EQCM can track the selectivity of ion adsorption. When the pH crosses the isoelectric point (pI) of the carbon electrode, EQCM data explicitly reveal a transition in the charge storage mechanism from anion adsorption/desorption to cation adsorption/desorption. This transition confirms the decisive role of the ionization state of surface functional groups in dictating ion‐selective adsorption [89]. Gao and co‐workers employed ac‐EQCM to investigate charge storage in electrochemically reduced graphene oxide(ERGO) electrodes with different oxygen functional group contents. The results showed that the charge‐compensation mechanism gradually shifted from anion‐dominated to cation‐dominated behavior as oxygen functionalities were removed. Ac‐EQCM further separated the contributions of hydrated cations, dehydrated cations, anions, and free water molecules, revealing distinct interfacial transfer kinetics for different species during electrochemical cycling [90].

EQCM data can be further exploited to quantitatively resolve real‐time ion fluxes and ionic currents. Using the deconvolution method described in Section 2.2.1, Lin et al. applied this method to analyze the electrochemical behavior of Ti_3_C_2_T*_x_* MXene supercapacitors in 1 mol/L H_2_SO_4_ electrolyte, calculating real‐time fluxes of H_2_O and H^+^ from EQCM data, as shown in Figure 7f. The J‐CV curve is highly symmetrical, confirming the excellent reversibility of the insertion/extraction process of H_2_O and H^+^, with the hydration of H^+^ significantly contributing to the double‐layer capacitance. The CV curve constructed based on the real‐time flux of H^+^ is shown in Figure 7f, where the calculated H^+^‐CV overlaps with the experimentally measured CV, confirming that H^+^ is the only charge carrier interacting with the Ti_3_C_2_T*_x_* electrode to shield its charge [50]. Niu et al. utilized EQCM to study the charging mechanism of conductive MOF electrodes in ionic liquids with different scan rates. By calculating the charging mechanism parameters based on the variation in the quantities of anions and cations, this phenomenon can be quantified. As shown in Figure 7g, at low scan rates (10 mV/s), the charging and discharging process is dominated by anion adsorption/desorption, whereas at high scan rates (1000 mV/s), ion exchange predominates. Further calculations of the ion fluxes and currents contributed by anions and cations, as shown in Figure 7g, reveal that the CV curves derived from the formulas for anions and cations are consistent with the experimental results [91].

#### 3.1.2 Evolution of Ion Solvation Structure

**FIGURE 8:**
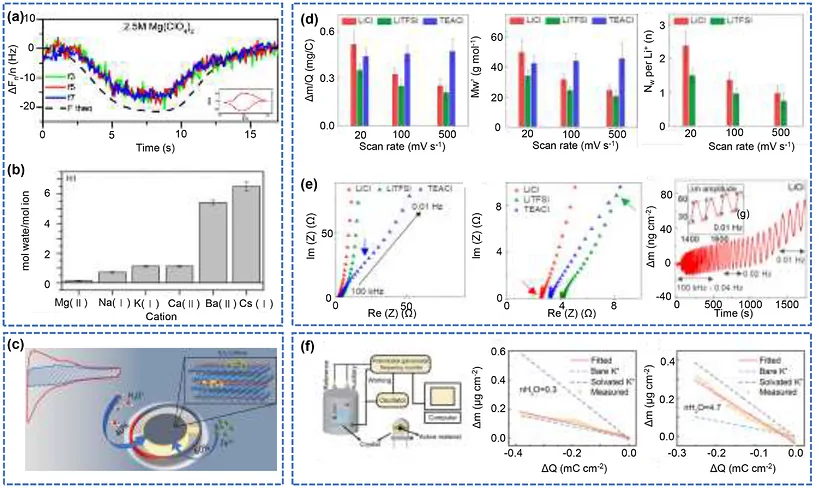
(a) Frequency changes recorded for the third, fifth, and seventh harmonics upon insertion/extraction of Mg^2+^ into the PI electrodes. The corresponding CV curves (scanned at 50 mV/s) are presented in the inset figures; (b) *χ*_w_ ratio calculated for the study system. Reproduced with permission [93]. Copyright 2021, American Chemical Society. (c) EQCM quantification diagram of the ratio of zinc ions to protons [95]; (d) Charge transfer mass change (Δ*m*/Δ*Q*) calculated from EQCM data in different electrolytes; Apparent molecular weight (*Mw*′) of transferred species; Number of water molecules (*N*_w_) transferred per embedded cation; (e) Frequency dependence and comparison of characteristic insertion time; Nyquist plot comparison for Ti_3_C_2_T*_x_* MLs in LiCl, LiTFSI, and TEACl; In situ EQCM‐D monitoring during EIS testing of Ti_3_C_2_T*_x_* MLs in LiCl. Reproduced with permission [96]. Copyright 2021, American Chemical Society. (f) EQCM experimental setup diagram; MMC curve and corresponding solvation number; Meso_C curve and corresponding solvation number. Reproduced under the terms of the Creative Commons CC‐BY 4.0 license [97]. Copyright 2025, Springer Nature.

The evolution of solvation structures and their desolvation dynamics at the electrode interface are fundamental scientific issues that determine the charge transfer kinetics, interface stability, and overall performance of EESDs. The concentration of the electrolyte affects the interfacial electrochemical behavior by regulating the ion‐solvent/ion‐ion interactions. Yang et al. used EQCM‐D to monitor the mass change of the Ca(BH_4_)_2_/THF electrolyte on a gold electrode at varying salt concentrations (0.5 to 1.5 m), revealing that as the salt concentration increased, solvated Ca^2+^ ions formed larger polymeric ion clusters. At an electrolyte concentration of 1.5 m, the solvation structure changes at different stages. These polymeric ions formed at the electrode interface influenced the calcium ion deposition and electrode reactions during the plating process [92]. Amey et al. employed EQCM‐D to systematically investigate the redox reactions and the resultant charge storage mechanisms of polyimide (PI) anodes in various aqueous electrolytes involving a range of monovalent and multivalent cations (Figure 8a). By quantitatively comparing the Faradaic charge and actual mass change rates, they calculated the number of water molecules (*χ*_w_) released for each cation embedded, as shown in Figure 8b. The *χ*_w_ value was significantly correlated with the solvation free energy (Δ*G_solvation_*) and effective radius (*R_eff_*) of the cation. Specifically, chaotropic cations with lower charge‐to‐radius ratios (e.g., Cs^+^) tend to shed a greater number of water molecules (*χ*_w _≈  6.51), a phenomenon that correlates with their accelerated redox kinetics. In contrast, strongly solvated kosmotropic cations (e.g., Mg^2+^) exhibit a *χ*_w_ value near zero (*χ*_w _≈  0.13), indicating that they are predominantly intercalated in a fully or partially solvated state. The EQCM‐D results revealed for the first time that the solvation properties of cations are key factors influencing the charge storage mechanisms and electrochemical performance of organic electrodes, providing molecular‐level insights into electrode design for high‐capacity aqueous multivalent‐ion batteries [93]. Meital et al. confirmed through EQCM that the pseudocapacitive process in the organic electrode PANI involved the co‐transport of ion embedding/extraction and water molecules (solvent). The degree of this solvent co‐transport was closely related to the PANI structure's pore space and rigidity [94].

The pore structure and surface chemistry properties of electrodes exert significant “squeezing” and “inductive” effects on the desolvation process. Zhang et al. employed EQCM to investigate the relationship between pore‐mouth size and solvation structure in hard carbon anodes for non‐aqueous sodium‐ion batteries using 1 m NaPF_6_ in EC/DEC‐based electrolytes. They found that carbon materials with relatively large pore mouths (e.g., activated carbon, AC) mainly accommodated solvent‐separated ion pairs (SSIPs), resulting in substantial solvent adsorption and the formation of thick organic‐rich SEI layers. In contrast, carbon molecular sieve materials with ultra‐small pore mouths (e.g., CMS‐1300) promoted stronger desolvation and favored anion aggregate (AGG)‐dominated solvation structures, which facilitated the formation of thinner inorganic‐rich SEI layers and improved interfacial Na^+^ transport kinetics [97]. Karol et al. used EQCM to explore the lithium insertion mechanism in layered V_2_O_5_ electrode materials, finding that the frequency change amplitude caused by unit charge transfer in V_2_O_5_‐12C‐DA was about an order of magnitude stronger than in V_2_O_5_‐12C‐DA. The estimated molar mass of the inserted species in V_2_O_5_‐12C‐DA was approximately 50 g/mol, significantly higher than the molar mass of desolvated Li^+^, consistent with the co‐insertion mechanism where each Li^+^ simultaneously inserts up to two electrolyte solvent molecules (EC/DEC). The EQCM measurements provided conclusive evidence for the solvent co‐insertion mechanism, supporting the idea that the nanoconfined geometry is a key factor in inducing the shift from solid solution insertion to solvent co‐insertion in lithium [98]. Yang et al. validated the desolvation behavior at the interface of multi‐channel porous carbon nanofibers through EQCM, quantitatively analyzing the charge storage carrier and solvation degree by comparing the experimental slope (*k*_e_) of the CV process with the theoretical slope (*k*_t_) derived from Faradaic calculations. This confirmed the key role of non‐local high charge–density sites in accelerating the desolvation process in the interface [99].

In the charge storage process of porous and layered electrode materials, ion (de)insertion is often not an independent process but is accompanied by the co‐migration of solvent molecules. EQCM provides invaluable experimental evidence for understanding the ion transport kinetics and solvation effects at the EEI and within pores. Gavriel et al. utilized EQCM to precisely decouple the complex charge storage mechanisms of Ti_3_C_2_T*_x_* MXene electrodes in aqueous electrolytes, particularly distinguishing the contributions of Zn^2+^ ions and H^+^ protons, as illustrated in Figure 8c. The study found that, in low‐concentration ZnCl_2_ electrolytes, the charge storage process was predominantly dominated by the insertion/extraction of H^+^·nH_2_O. In high‐concentration ZnCl_2_, however, Zn^2+^ ions, in a partially desolvated form, became the main charge carriers, highlighting the role of electrolyte concentration in regulating the solvation/desolvation energy barrier of ions. This provided key experimental evidence for electrolyte optimization in high‐capacity ion storage devices [95]. Yun et al. used EQCM‐D to explore the effect of anion characteristics and time scales on the control of cation insertion storage mechanisms. The results indicated that, although anions do not directly insert into the MXene layers, their chemical structure significantly affects the hydration state and insertion kinetics of cations. As shown in Figure 8d, in the LiCl electrolyte, Li^+^, carrying more water molecules, exhibited the highest area capacitance and fastest characteristic insertion time. In LiTFSI, however, the asymmetric TFSI^−^ anion might weaken the hydration state and insertion efficiency of Li^+^ by altering the local electrostatic environment or spatial steric hindrance, leading to reduced capacitance. Combining with EIS frequency‐domain analysis, as shown in Figure 8e, EQCM‐D further revealed the frequency dependence of the ion insertion process, providing important in situ experimental evidence for understanding the structural‐dynamics‐performance relationships in MXene‐based energy storage materials [96]. Akshay et al. used real‐time mass tracking data from EQCM to reflect the insertion behavior of MXene with different nanostructure sizes. After size reduction, the mass exchange increased, and the mass change for miniaturized MXene was greater than the theoretical mass calculated based on the electrochemical exchange of bare Li^+^, suggesting that water molecules are exchanged with hydrated Li^+^ cations and/or free‐form Li^+^ ions. This revealed, at the molecular level, the intrinsic impact of material layer number and structural scale changes on ion transport dynamics and interface behavior [100].

The quantitative analysis capability of EQCM can be used to elucidate the mechanisms of multi‐species cooperative insertion during electrochemical processes. Deng et al. used EQCM to investigate the desolvation process of solvated ions in electrodes with different pore sizes in aqueous potassium‐ion batteries, as shown in Figure 8f. The Δ*m*–Δ*Q* analysis showed that the solvation number for the smaller‐pore MMC was 0.3, while for large‐pore Meso_C, it was 4.7. After introducing 4‐aminobenzo‐18‐crown‐6 (18C) into Meso_C via EDC‐mediated amidation, the solvation number of Meso_18C decreased to 2.3, indicating that the introduction of 18C significantly enhanced the desolvation of hydrated potassium ions. This highlights how appropriate pore design can effectively control the desolvation process and improve electrochemical performance [101]. Hu and co‐workers employed water‐rich K_0.01_Mn[Cr(CN)_6_]_0.74_·4.75H_2_O (MnHCC) as an anode in a highly concentrated aqueous electrolyte (21 m KOTF) to investigate the role of interstitial water in potassium‐ion storage. EQCM analysis revealed that the intercalated charge carriers consisted of mixed solvated species, including K^+^‐2H_2_O and K^+^‐H_2_O, and that the observed mass evolution was closely associated with the migration and de‐intercalation of interstitial water within the MnHCC framework during cycling. It should be noted that the original article and its Supporting Information did not explicitly report motional resistance (*R*_m_) or dissipation‐factor data to independently verify the rigid‐film/gravimetric condition required for the Sauerbrey analysis. Therefore, the EQCM‐derived mass changes should be interpreted under the Sauerbrey assumption and in conjunction with the complementary FTIR, XRD, GITT, GEIS/DRT, and AIMD results. These combined results indicate that dehydration of interstitial water increases ion‐diffusion barriers and structural distortion, resulting in enhanced charging polarization. After introducing dihydroxyacetone (DHA) as an electrolyte additive, the formation of K^+^‐DHA complexes effectively suppressed the dehydration process, alleviated framework distortion, and stabilized the electrochemical reaction kinetics [102]. Lu and co‐workers, in water‐based vanadium hexacyanoferrate materials, measured an equivalent insertion molar mass of 84.6 g/mol, significantly higher than the mass of bare Mn^2+^ ions (55 g/mol), confirming the co‐insertion of Mn^2+^ with partially hydrated or H^+^ species [103].

#### 3.1.3 Analysis of Multi‐Process Charge Storage Mechanisms

**FIGURE 9:**
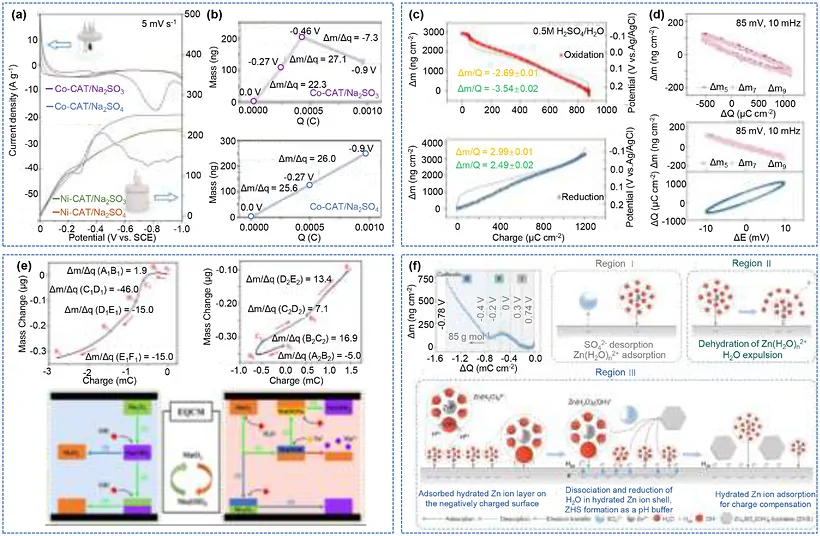
(a) CV curves of Co‐CAT/Na_2_SO_3_, Ni‐CAT/Na_2_SO_3_, Co‐CAT/Na_2_SO_4_, and Ni‐CAT/Na_2_SO_4_ electrodes at a scan rate of 5 mV/s (top) and their corresponding EQCM mass change curves (bottom); (b) Mass change versus Coulomb number for electrodes within the potential range from −0.9 to 0 V (vs. SCE). The slopes (Δ*m*/Δ*Q*) are marked and converted to g/mol/e. Reproduced with permission [104]. Copyright 2022, John Wiley and Sons. (c) Relationship between mass change and charge during the redox process; (d) Relationship between mass change and charge during a sinusoidal period, with a 10 mV amplitude. Reproduced with permission [105]. Copyright 2023, Elsevier. (e) Δ*m* curves during charging and discharging. Schematic diagram of the redox conversion mechanism of Al‐MnO_2_ electrodes in alkaline electrolyte, showing key reaction pathways and structural changes. Reproduced with permission [106]. Copyright 2025, John Wiley and Sons. (f) Relationship between electrode Δ*m* and Δ*Q* during cathodic scanning in cyclic voltammetry, with arrows indicating polarization direction and three different regions marked in different colors. Schematic diagram of charge storage processes in different potential regions (Regions I, II, and III). Reproduced under the terms of the Creative Commons CC‐BY 4.0 license [107]. Copyright 2025, John Wiley and Sons.

Redox reactions are central to the energy storage processes of pseudocapacitive materials and battery‐type electrodes. Understanding their reaction pathways, intermediate products, and accompanying ion transport behavior is crucial for optimizing material performance. EQCM can precisely identify the dominant reactants (such as cation insertion, anion redox, or adsorption behavior) within different potential ranges and reveal the profound effects of material structural modifications (such as pre‐insertion and crystallization water regulation) on the reaction pathways and reversibility. Cheng et al. used EQCM to analyze the redox processes of typical two‐dimensional conductive MOFs (Co‐CAT and Ni‐CAT) in Na_2_SO_3_ electrolyte, as shown in Figure 9a,b. Based on the potential ranges of the redox reactions, three regions can be distinguished. In Region I, the mass increase per electron (Δ*m*/Δ*Q*) for Co‐CAT/ Na_2_SO_3_ and Co‐CAT/Na_2_SO_4_ was 22.3 and 25.6 g/mol, respectively. This is close to the theoretical Δ*m*/Δ*Q* of 23 g/mol per electron for the C = O to C‐O transformation in the CAT ligand, indicating that the insertion of Na^+^ ions caused the mass increase. In Region II, for Co‐CAT/Na_2_SO_3_, Δ*m*/Δ*Q* was 27.1 g/mol, which closely matches the theoretical value of 28 g/mol for the SO_3_^2−^ to S_2_O_3_^2−^ redox reaction. For Co‐CAT/Na_2_SO_4_, Δ*m*/Δ*Q* was 26 g/mol, possibly related to the adsorption of SO_4_^2−^ ions. In Region III, for Co‐CAT/Na_2_SO_3_, Δ*m*/Δ*Q* was −7.3 g/mol, which is close to the theoretical value of −10 g/mol for the S_2_O_3_^2−^/S^2−^ redox reaction [104]. Ma et al. introduced EQCM‐D into polymer air batteries and measured the charge transfer species in the redox process. Specifically, in the first and second oxidation reactions, water and hydrogen ions transferred 6.18 and 8.46 water molecules, respectively, while in the first and second reduction reactions, they transferred 6.96 and 5.62 water molecules, as shown in Figure 9c. To elucidate the dynamic mass insertion/extraction process, in situ EQCM‐D combined with EIS was employed. A 10 mV sinusoidal potential perturbation was applied to the poly(benzimidazobenzophenanthroline)‐coated quartz crystal, while the corresponding frequency and dissipation responses were simultaneously recorded. Figure 9d shows the relationship between Δ*m* and Δ*Q*, and the relationship between Δ*Q*, Δ*m*, and Δ*E*, both of which display characteristic elliptical tilts, corresponding to Lissajous Figures indicating phase shifts. Comparing the Δ*Q*–Δ*m*–Δ*E* responses allows for qualitative determination of whether cations or anions are involved in transfer at a given EIS frequency [105]. Cheng et al. used in situ EQCM to reveal the multi‐step reaction mechanisms of manganese dioxide in alkaline aqueous electrolytes, providing fundamental insights into the redox mechanism of manganese dioxide. The redox behaviors of Al‐MnO_2_ and Na‐MnO_2_ in 1 m NaOH electrolyte are shown in Figure 9e. The study found that Al‐MnO_2_, which contains less crystallization water than Na‐MnO_2_, significantly altered the reaction pathway of MnO_2_. Pre‐insertion of Al^3+^ ions increased the contact area between Mn^2+^ and OH^−^ ions in the electrolyte, promoting the formation of Mn(OH)_2_. This process effectively inhibited the dissolution of Mn^2+^ ions into the electrolyte, preventing the loss of active electrode material [106].

EQCM can effectively decouple the synergistic transport behavior of various ions and solvent molecules in complex electrolyte systems, revealing the species evolution at the molecular level during the electrochemical process at the electrode. For example, in aqueous zinc‐ion hybrid capacitors, the charge storage mechanism becomes difficult to analyze due to the involvement of both double‐layer capacitance and surface redox reactions. EQCM offers an intuitive and straightforward analysis method that can rapidly identify the charge carriers involved in the storage process. Li et al. monitored the charge storage mechanism of mesoporous carbon in 2 mol/L ZnSO_4_ electrolyte using EQCM, as shown in Figure 9f. The summary of the analysis is as follows: in the high potential range (Region I), the primary processes were the adsorption and desorption of water, zinc ions, and SO_4_^2−^ ions, exhibiting obvious capacitive behavior. In the middle potential range (Region II), the desolvation of Zn^2^^+^ ions further increased the capacitance. In the high cathodic polarization range (Region III), charge storage occurred through the reduction of water molecules to form surface‐adsorbed H^+^ and OH^−^ ions, as well as the formation of zinc hydroxysulfate hydrates. This indicates that the charge storage mechanism in zinc‐ion hybrid capacitors is a multi‐stage process, with the reverse process occurring during anodic polarization [107]. The power of ac‐EQCM in deconvoluting complex charge‐storage mechanisms is exemplified by the study of Li‐birnessite type MnO_2_, where it was used to distinguish between pure intercalation and surface adsorption processes. By coupling fast QCM with electrochemical impedance spectroscopy, researchers quantitatively identified the simultaneous but distinct dynamic contributions of alkali cations (Li^+^ or Na^+^), their hydrated counterparts, and free water molecules. Crucially, the technique detected an opposite flux direction of free water molecules relative to the cations. This diagnostic signature experimentally demonstrates the desolvation effect, where a population of hydrated ions sheds a portion of their hydration shell prior to or during transfer at the electrode/electrolyte interface. Such dynamic insights allow for the separation of Faradaic and non‐Faradaic contributions, providing a level of mechanistic detail that conventional EQCM cannot achieve [59]. Wang et al. used EQCM to examine the coordination species in the discharge process of a water‐based zinc‐ion battery using 4‐fluorosalicylic acid (F‐H_2_SA)‐modified MnO_2_ as the cathode material. Based on mass change data, the reduction process could be divided into two stages. From 1.8 to 1.5 V, the mass increase was 0.160 mg/C, which is between the masses of H^+^ (0.010 mg/C) and H_3_O^+^ (0.197 mg/C), indicating the synergistic coordination of H^+^ and H_3_O^+^ ions. From 1.45 to 1.3 V, the electrode mass change sharply increased to 0.454 mg/C, which closely matches the mass of hydrated H^+^ and Zn^2+^. As the discharge continued to 1.3 V, the mass further increased to about 0.712 mg/C, closely matching the mass of Zn^2+^·4H_2_O. The mass increase was primarily attributed to cation coordination [108]. Similarly, Zhang et al. used EQCM to verify that the δ‐MnO_2_ electrode with intercalated metal ions in a water‐based zinc‐ion battery participated in different charge storage carriers during different stages of the discharge process [109]. Kang et al. used EQCM‐D to track real‐time mass changes during the charge and discharge processes of dihydro‐octaaza‐pentacene (DOP) cathodes, revealing the multi‐electron, dual‐active‐center charge storage mechanism [110]. Wang and co‐workers used EQCM to quantify the storage process of Zn^2+^/H^+^ in a water‐based zinc‐ion battery during discharge and found that the charge carriers were 2Zn(2H_2_O)^2+^ and 8H_3_O^+^, indicating that H^+^ storage dominated [111].

### 3.2 Electrode Structural Evolution

Electrode materials undergo complex structural evolution during charging and discharging processes, particularly in terms of the chemical and physical changes at the EEI. Through real‐time monitoring of mass and dissipation via EQCM, the microscopic morphology of the electrode can be organically linked to the macro performance evolution. This not only reveals the structural stability of electrode materials but also precisely identifies the potential ranges at which failure occurs, highlighting the key factors responsible for performance degradation. This provides targeted optimization guidelines for material design and offers crucial experimental evidence for developing high‐performance, high‐stability energy storage systems.

#### 3.2.1 Interfacial Regulation

**FIGURE 10:**
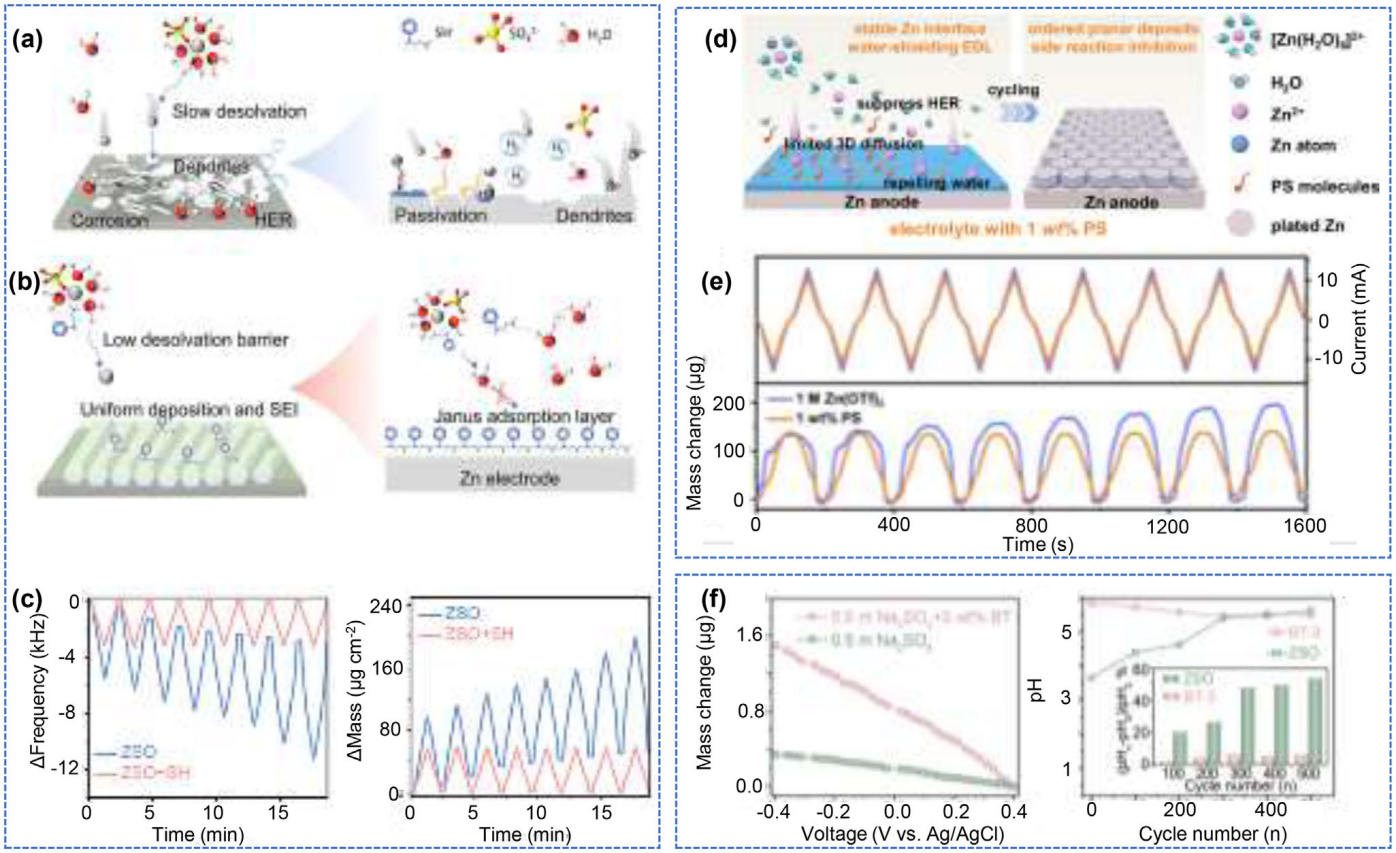
(a) Schematic of Zn deposition behavior in ZSO and (b) ZSO + SH electrolytes; (c) Frequency and corresponding mass change curves from EQCM in different electrolyte systems. Reproduced with permission [113]. Copyright 2026, John Wiley and Sons. (d) Effect of PS molecules on electrolyte and interfacial chemistry in 1 m Zn(OTf)_2_ and electrolyte containing 1 wt.% PS; (e) EQCM‐based chronocoulometry and mass changes at a scan rate of 10 mV/s in different electrolytes. Reproduced under the terms of the Creative Commons CC‐BY 4.0 license [115]. Copyright 2025, Springer Nature. (f) EQCM mass changes and pH variation of electrolyte during LSV testing. Reproduced with permission [116]. Copyright 2024, John Wiley and Sons.

The behavior of electrode interfaces and their regulation mechanisms are key factors in enhancing the performance of energy storage devices. EQCM can precisely quantify the mass evolution at the electrode interface, offering insights not only into the competitive adsorption behavior of additives on the electrode surface, but also into the microscopic regulation of solvent structure and interface phase transition dynamics. These capabilities provide critical guidance for interfacial engineering. For example, in aqueous zinc‐ion batteries, uneven zinc deposition on the negative electrode remains a major issue limiting practical applications. Electrolyte additive engineering has proven to be an effective strategy for regulating the interfacial microenvironment and controlling the Zn^2+^ nucleation/deposition dynamics [112]. Zhang and co‐workers introduced sodium hippurate (SH) as an additive into an aqueous zinc sulfate electrolyte, as shown in Figure 10a,b. The mass change on the electrode surface exhibited extremely high reversibility(Figure 10c), indicating that SH forms a Janus adsorption interface, which regulates the solvent structure and deposition behavior of zinc, effectively inhibiting side reactions and enabling reversible and uniform zinc deposition [113]. Similarly, Pan et al. used the unique zwitterionic structure of trimethylamine N‐oxide (TMAO) to construct a stable zinc ion‐rich protective layer (TMAO‐Zn), which promoted uniform zinc deposition and reduced corrosion. EQCM data showed that the mass efficiency of zinc metal with TMAO‐Zn added increased to over 99.8%, confirming that the TMAO‐Zn protective layer effectively reduced concentration polarization and suppressed side reactions [114]. Additionally, Peng et al. used polysorbate (denoted as PS) as a functional additive to construct an organic–inorganic hybrid interfacial film. As shown in Figure 10d,e, EQCM results indicated that the mass efficiency of the electrolyte containing 1 wt.% PS consistently remained above 99.8%, outperforming the 1 m Zn(OTf)_2_ electrolyte, confirming that PS molecules enhanced the deposition/stripping reversibility over a wide potential range [115].

The imbalance between ion diffusion in the double layer and electron transfer is a key factor in dendrite formation. The preferential adsorption of additives to exclude interfacial water molecules is an effective way to create a “waterless” Helmholtz plane (IHP). EQCM can quantitatively monitor the effects of different additives on the mass of surface‐adsorbed species, thus revealing their regulation of the double‐layer structure. Zheng et al. added dimethyl biguanide hydrochloride (MF·HCl) as an additive to a ZnSO_4_ electrolyte to achieve competitive adsorption with water. Through EQCM, it was confirmed that the zinc negative electrode preferentially exchanges electrons with MF^2+^, providing a solid foundation for interfacial charge regulation through stable adsorption [117]. Li et al. used bis(2‐hydroxyethyl)amino‐tris(hydroxymethyl)methane (BT) as an additive to achieve three types of regulation. BT was shown to regulate the pH, as illustrated in Figure 10f, lowering the hydrogen evolution reaction (HER) overpotential. Additionally, BT molecules, which contain multiple hydroxyl functional groups, preferentially adsorbed on the zinc negative electrode, reducing the water molecules in the double layer. Furthermore, BT participated in solvent structure regulation and preferential reduction, forming a nitrogen‐containing SEI and promoting uniform zinc deposition [116]. Bu et al. used N,N‐bis(2‐hydroxyethyl)glycine (BHEG) to construct an adsorption layer in a water‐based zinc‐iodine battery, and EQCM quantitatively demonstrated that its “water barrier” effect helped buffer the interface pH and suppress hydrogen evolution reactions [118]. Duan et al. quantitatively monitored the adsorption of different amino acids at the interface using EQCM and found that succinamide acid (SuaA) induced electric double layer (EDL) reconstruction through strong adsorption, confirming the key role of carboxyl groups in the surface EDL rebuilding process [119].

The electronic structure and physicochemical environment at the electrode surface can be precisely optimized by constructing heterogeneous interfaces, surface modification, or ion doping, which fundamentally enhances the kinetic response. Cai et al. constructed a TiO_2_/TiSe_2_‐C heterostructure interface with built‐in electric fields. Through EQCM monitoring, they found that the intercalated species transitioned from [Zn(H_2_O)_6_]^2+^ to [Zn(H_2_O)_2_]^2+^, significantly accelerating ion transport kinetics. This indicates that the heterostructure can regulate the solvation sheath of zinc ions, thus enhancing ion transport kinetics and reducing structural changes during insertion/extraction processes, ensuring high rate and long‐cycle electrochemical performance of the anode material in deep discharge states [120]. Jin et al. constructed a ferrocyanide “skin” on the surface of transition metal oxides, which effectively mitigates severe self‐discharge caused by interfacial side reactions, particularly the oxygen evolution reaction (OER). EQCM real‐time monitoring revealed a significant slowing down of mass loss during the self‐discharge process, proving that the novel coordination interaction between NiO and Fe(CN)_6_^4−^ at the interface effectively mitigates the severe dissolution of NiOOH to Ni(OH)_2_ during electrochemical and open‐circuit potential processes [121]. Xu et al. used a strategy of co‐doping cations and anions to modulate the electronic structure of oxygen‐active centers and ion adsorption energy. By co‐doping Ca/N in δ‐MnO_2_ (Ca/N‐MnO_2_), they adjusted the *ε*_p_ orbitals of oxygen and the d‐band center of manganese (*ε*_d_), and EQCM confirmed the enhanced adsorption capacity and rate of Zn^2+^/H^+^ ions at the interface [122]. Xue et al. used EQCM to monitor the real‐time mass change of co‐doped polymer electrodes and quantified the blocking effect of auxiliary dopants (PO_4_^3−^) on the outward migration of active species. This directly confirmed that this interfacial regulation strategy could effectively stabilize the polymer matrix structure, significantly improving the reversible capacity and cycling performance of polymer electrodes [123].

#### 3.2.2 Failure Mechanisms

**FIGURE 11:**
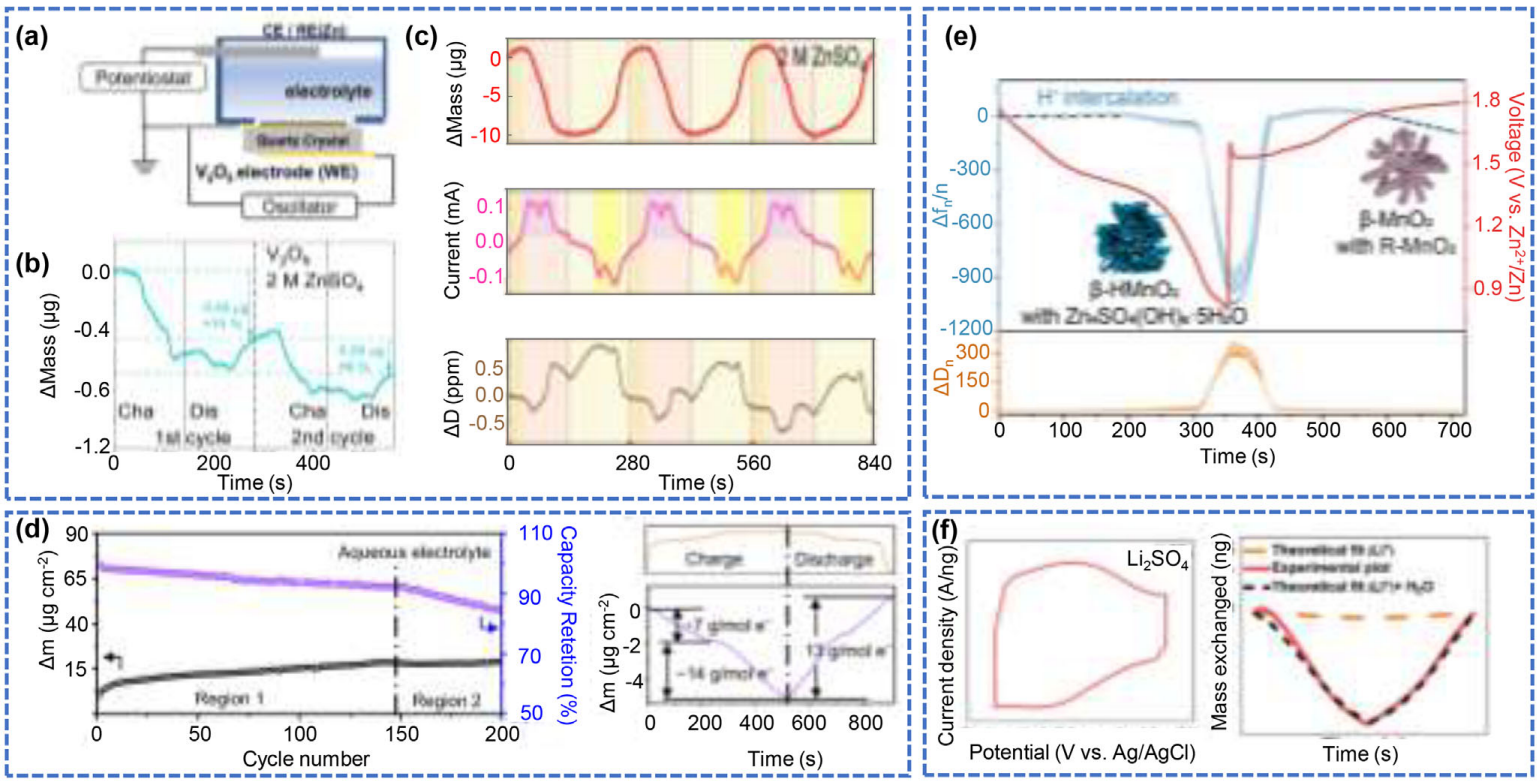
(a) Schematic of the EQCM‐D setup; (b) Mass change of commercial V_2_O_5_ in 2 m ZnSO_4_ electrolyte during EQCM‐D cycling tests; (c) Time‐dependent behavior of VOP electrode during EQCM‐D testing, scan rate 10 mV/s, (i) Mass, (ii) Current, (iii) Dissipation factor, all plotted versus time. Reproduced with permission [124]. Copyright 2025, American Chemical Society. (d) EQCM test results of LMO electrode in 1 m Li_2_SO_4_ electrolyte during cycling, and mass changes during LMO charging and discharging. Reproduced under the terms of the Creative Commons CC‐BY 4.0 license [127]. Copyright 2023, Springer Nature. (e) In situ EQCM‐D analysis of β‐MnO_2_, including the voltage‐profile‐correlated changes in frequency and dissipation during the 10th cycle, where the black dashed line represents the theoretical frequency variation calculated based on the Sauerbrey‐Faraday equation. Reproduced with permission [129]. Copyright 2024, American Chemical Society. (f) CV measurements and corresponding mass exchange profile procured from EQCM measurements. Reproduced under the terms of the Creative Commons CC‐BY license [130]. Copyright 2024, John Wiley and Sons.

EQCM‐D enables the monitoring of electrode health across various charging, cycling, and aging conditions. By retrieving the electrode's thickness‐modulus distribution, it facilitates the simultaneous monitoring of mechanical properties and mass changes. This provides experimental foundations and evaluation standards for developing high‐stability electrodes. Li et al. used in situ EQCM‐D to study the failure mechanisms of vanadium oxide in aqueous zinc‐ion batteries. The EQCM‐D testing setup is shown in Figure 11a. The results revealed that in acidic conditions, vanadium oxide primarily undergoes an H^+^‐dominated intercalation mechanism, leading to OH^−^ formation at the electrode–electrolyte interface, which causes vanadium oxide dissolution and capacity decay, as shown in Figure 11b. To suppress dissolution, nanosheet‐structured V_2_O_5_@PEDOT (VOP) was prepared by in situ polymerization of 3,4‐ethylenedioxythiophene (EDOT) and exfoliation of commercial micrometer‐sized V_2_O_5_ into nanosheets. The VOP test results are shown in Figure 11c. Unlike commercial V_2_O_5_, VOP exhibited reversible mass changes during oxidation and reduction, indicating that PEDOT modification can effectively inhibit dissolution. These results provide guidance for improving battery performance and cycling stability [124]. Huo and co‐workers employed EQCM to reveal the failure mechanisms of vanadium nitride (VN) electrodes in alkaline electrolytes. Combined with cyclic voltammetry (CV), they found irreversible mass increases (+151.3 ng) and decreases (−60.9 ng) in the −0.4 to 0 V potential range, indicating an irreversible oxidation reaction in this range [125]. Sun et al. used EQCM to study the impact of electrode surface composition on the double‐layer structure and capacitance performance in aged acetonitrile‐based supercapacitors. They found that the negative shift of the zero charge potential (PZC) of aged electrodes triggered specific adsorption of cations. This specific adsorption also occupied the IHP, leading to an increased EDL thickness and a decrease in capacitance. Meanwhile, cationic side reactions occurred at the negative electrode, causing a sharp decline in negative electrode capacitance. Due to electrode aging, the charge storage mechanism also changed during cycling, with ion exchange replacing ion adsorption under negative polarization, which was the main cause of the negative electrode capacitance decline [126]. Huang et al. used EQCM to reveal the failure mechanisms of LiMn_2_O_4_ (LMO) positive electrodes in aqueous electrolytes. The EQCM results provided key evidence for the failure mechanism of LMO, showing that water molecules combined with protons to form H_3_O^+^ and intercalated into the LMO lattice, as shown in Figure 11d. The massive intercalation of H_3_O^+^ blocked the Li^+^ diffusion channels, leading to a sharp decline in Li^+^ transport kinetics. Additionally, instability caused by intercalation led to the dissolution of Mn^2+^ into the electrolyte, further accelerating capacity decay [127].

The dissipation factor (Δ*D*) monitored by EQCM‐D is directly related to the structural rearrangement and softening of the electrode interface. When the charge storage process exhibits high reversibility and stability, the Δ*D* change is minimal. In contrast, unstable ion insertion/extraction leads to severe swelling and structural disorder in the polymer film, resulting in a significant increase in Δ*D*. Collaborative analysis of the gravimetric and dissipation data measured by Ma et al. reveals that chaotropic anions effectively modulate the water activity and solvation structure within the polymer backbone. This modulation optimizes the ion transport channels and maintains minimal viscoelastic fluctuations during the charging/discharging cycles. Significant differences in the number of water molecules accompanying different ions (Cl^−^, BF_4_^−^, and OTf^−^) during insertion were observed, with Cl^−^ accompanying 30‐38 water molecules, leading to severe polymer swelling and capacity loss. The Δ*D* monitoring directly correlated with the viscoelastic changes of the polymer framework and revealed that the Hofmeister effect in the electrolyte significantly influenced ion dynamics and structural stability by regulating the solvation structure within the polymer [128]. Wu et al. investigated the failure mechanisms of β‐MnO_2_ in aqueous zinc‐ion batteries (Figure 11e), finding that when Zn_4_SO_4_(OH)_6_·nH_2_O (ZHS) and other by‐products formed on the electrode surface, the dissipation value significantly increased, indicating the formation of a loose, low‐conductivity layer on the electrode surface. This layer hindered further ion transport, leading to increased electrochemical polarization and eventually triggering battery failure [129]. Akshay et al. analyzed the performance degradation and failure of functionalized graphene derivatives in aqueous supercapacitors (Figure 11f). EQCM results showed that ions carry a substantial number of solvated water molecules during insertion, and repeated swelling and contraction caused the graphene layers to stack or the active functional groups to peel off. The Δ*D* indicated a transition from rigidity to viscoelasticity in the electrode layers, suggesting a loosening of the physical structure. They also used EQCM to verify the impact of pre‐stored water molecules in nano‐confining spaces on cycling stability, finding that an appropriate amount of water assisted ion transport, while excessive confined water during cycling exacerbated gas evolution side reactions. Abnormal mass fluctuations and increased dissipation, as observed in EQCM, intuitively revealed how tiny gas bubbles generated by water electrolysis block ion channels [130].

### 3.3 Deposition Behavior at the Solid–Liquid Interface

The chemical instability of the EEI and the phase change behavior during metal deposition/stripping processes induce continuous reconstruction of the interface morphology, uniformity, and chemical composition. This complex interface evolution often accompanies dendrite growth, parasitic side reactions, and unstable SEI, which severely limit the cycle life and safety performance of batteries [131]. A deep understanding of the intrinsic relationship between deposition morphology, interface chemistry, and electrochemical reversibility is crucial for electrolyte engineering and interface control. EQCM plays a vital role in analyzing the solid–liquid interface deposition behavior at metal electrodes and electrolyte interfaces, particularly in understanding the formation of SEI or CEI (counter‐electrode interface) and the metal deposition process. Its core value lies in the real‐time, quantitative tracking of nanoscale changes in the electrode surface mass and viscoelasticity, thereby revealing the dynamic formation mechanisms of SEI (CEI) films, the evolution of chemical components, and the physicochemical characteristics of deposition products.

#### 3.3.1 Nucleation Effect Derived From Electrolytes

**FIGURE 12:**
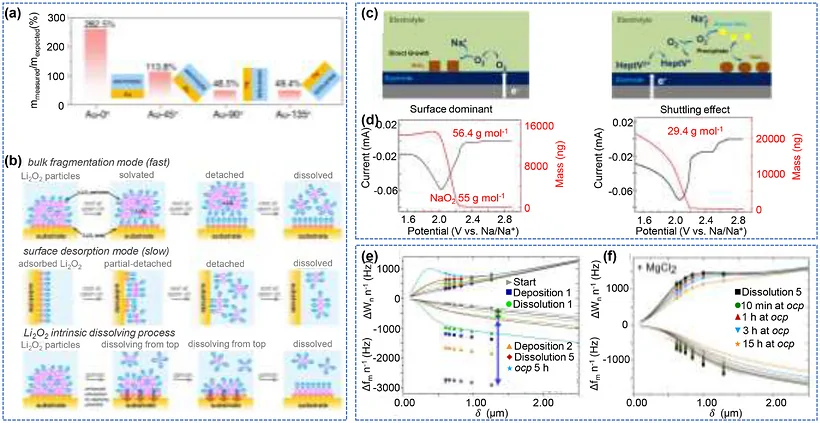
(a) Ratio of measured mass deposition to expected mass deposition on electrodes at different angles; (b) Schematic of various dissolution processes corresponding to EQCM results. Reproduced under the terms of the Creative Commons CC‐BY 3.0 license [132]. Copyright 2025, Royal Society of Chemistry. (c) Schematic of HeptVBr_2_ shuttle effect on NaO_2_ formation; (d) Mass changes for systems with and without 5 mm/L HeptVBr_2_ additives. Reproduced with permission [134]. Copyright 2021, Elsevier BV. (e) Mg(TFSI)_2_ electrolyte undergoing 5 plating/stripping cycles at ±1 mA/cm^2^ current density (90 s per cycle) and selected fluid dynamics spectra after 5 h of open‐circuit potential (OCP) exposure; (f) Mg(TFSI)_2_/MgCl_2_ electrolyte undergoing 5 plating/stripping cycles at ±500 µA/cm^2^ current density (3 min per cycle) and after 10 min, 1 h, 3 h, and 15 h of OCP exposure, with corresponding uniform porous layer model fitting curves. Reproduced under the terms of the Creative Commons CC‐BY‐NC‐ND license [135]. Copyright 2024, John Wiley and Sons.

In alkali metal‐gas battery systems, the nucleation, deposition, and dissolution dynamics of discharge products (such as Li_2_O_2_ or Li_2_CO_3_) are core factors that determine the energy efficiency and cycling stability of the battery. EQCM technology can quantitatively identify the growth pathways and dissolution modes of the products, providing key experimental data for optimizing porous electrode structures and selecting efficient redox mediators. He et al. used EQCM to study and quantify the deposition and dissolution dynamics of Li_2_O_2_, revealing that the electrode orientation greatly influences the formation pathway and deposition amount of Li_2_O_2_, as shown in Figure 12a. Additionally, two dissolution modes of Li_2_O_2_ on the electrode surface were identified: surface dissolution and bulk fragmentation, as shown in Figure 12b. The dissolution rate for surface dissolution is relatively slow (3.2 ng/cm^2^/min), as it requires solvent molecules to penetrate the passivation layer, while bulk fragmentation occurs much faster (300 ng/cm^2^/min), with solvent molecules inserting between the Li_2_O_2_ (ads) | Li_2_O_2_ (bulk) interface, causing the bulk Li_2_O_2_ to quickly detach from the electrode surface. Therefore, the choice of solvent is critical for the dissolution process. This work demonstrates that solving the dissolution issue of Li_2_O_2_ can enable high practical energy densities for Li–O_2_ batteries [132]. Hu et al. introduced the redox mediator LiI into Li‐CO_2_ batteries to achieve high energy efficiency and cycling stability. Using EQCM, they revealed the role mechanism of LiI in enhancing the reversibility of Li–CO_2_ batteries. Mass changes indicated that the MPE value had a consistent slope of approximately 40 g/mol during discharge, showing that the decomposition and deposition of Li_2_CO_3_ during charging and discharging were reversible, with the redox mediator significantly enhancing the reversibility of the homogeneous reaction [133]. Yang et al. used EQCM to study the effect of redox control agents (HeptVBr_2_) on the reaction mechanism in Na‐O_2_ batteries. As shown in Figure 12c, the results showed that in the absence of additives, the calculated average molecular weight of the product was about 56.4 g/mol, which is very close to the theoretical molecular weight of NaO_2_, indicating that the reaction was surface‐controlled. In contrast, with the addition of HeptVBr_2_, the mass change was only 29.4 g/mol, indicating a change in the reaction pathway. As shown in Figure 12d, HeptVBr_2_ acts as a “redox mediator” that transfers electrons from the electrode surface to the electrolyte. This process facilitates the solution‐phase formation of NaO_2_ rather than its localized deposition on the electrode surface, thereby optimizing the reaction kinetics and significantly enhancing the cell capacity [134].

The reversible electroplating/stripping behavior of metal anodes (such as Zn and Mg) is critical to achieving high‐performance aqueous or multivalent metal batteries. EQCM‐D, by analyzing the synchronized evolution of frequency and dissipation, can not only differentiate between dense and loose deposition layers but also quantitatively analyze the role of additives or electrolyte components in regulating interface stability, deposition morphology, and cycling reversibility. Zhang et al. used real‐time frequency (*f*_3_) and dissipation (D_3_) changes during the zinc plating/stripping processes to study the interface chemistry of zinc electrodes in different electrolytes. When zinc deposition on the crystal substrate appeared loose and irregular during oscillation, it led to a shorter decay time, accompanied by higher dissipation. In contrast, dense and uniform zinc deposition layers corresponded to longer decay times and lower dissipation. The synergistic effect of diethyl carbonate (DEC) and ethyl acetate (EA) promoted the uniform deposition of zinc on the electrode [136]. Zhang et al. combined CV testing to monitor the real‐time mass changes during zinc deposition and stripping processes on the electrode. In ZnSO_4_ electrolyte, the mass efficiency (ME) was only 48.3% on average, indicating significant side product deposition and active zinc loss. However, after introducing g‐C_3_N_4_ nanosheets, ME increased to 98.5%, and coulombic efficiency (CE) reached 99.6%, showing that side reactions were significantly suppressed. EQCM quantitative analysis showed that g‐C_3_N_4_ nanosheets were embedded in the zinc deposition layer during the deposition process, accounting for about 3.28% of the mass, which helped form a dense, vertically aligned zinc deposition structure and improved cycling stability [137]. Benjamin W et al. quantitatively monitored the Mg plating/stripping process in magnesium batteries, as shown in Figure 12e,f. For electrochemically stable Mg[B(HFIP)_4_]_2_ electrolytes, the results showed highly reversible Mg deposition and stripping, indicating no significant passivation layer formed at the interface. For the less stable Mg(TFSI)_2_ electrolyte, a continuous and significant mass deposition was observed during reduction, with a large change in Δ*D* signal, directly confirming the electrolyte component decomposition and the formation of a loose and unstable passivation layer, leading to low Coulombic efficiency. By introducing MgCl_2_ as an additive, the results showed a significant change in the passivation layer evolution path. In the unstable Mg(TFSI)_2_ system, the presence of Cl^−^ facilitated the formation of a thin and dense passivation layer. EQCM‐D, through its simultaneous monitoring of mass and viscoelasticity, provides irreplaceable quantitative and real‐time evidence for understanding the formation dynamics, composition, structure of passivation layers, and the regulation mechanism of electrolyte components under operational conditions in Mg batteries [135].

#### 3.3.2 Formation and Evolution of Interfacial Phases

The SEI is a passivation layer that forms spontaneously during electrochemical cycling. An ideal SEI should possess both chemical stability to suppress ongoing side reactions and mechanical stability to withstand the volume changes of the electrode [138]. Since the interphase layer forms on both the anode and cathode, these are referred to as the SEI on the anode side and the cathode electrolyte interphase layer (CEI) on the cathode side [139]. EQCM translates intricate interfacial evolution into quantifiable mass fluxes, while its dissipation factor enables the identification of the mechanical integrity and dynamic dissolution behaviors of the SEI and CEI. Consequently, this approach unveils the regulatory mechanisms of electrolyte components on the passivation efficacy, solubility, and viscoelastic footprints of these interphases at a molecular level.

**FIGURE 13:**
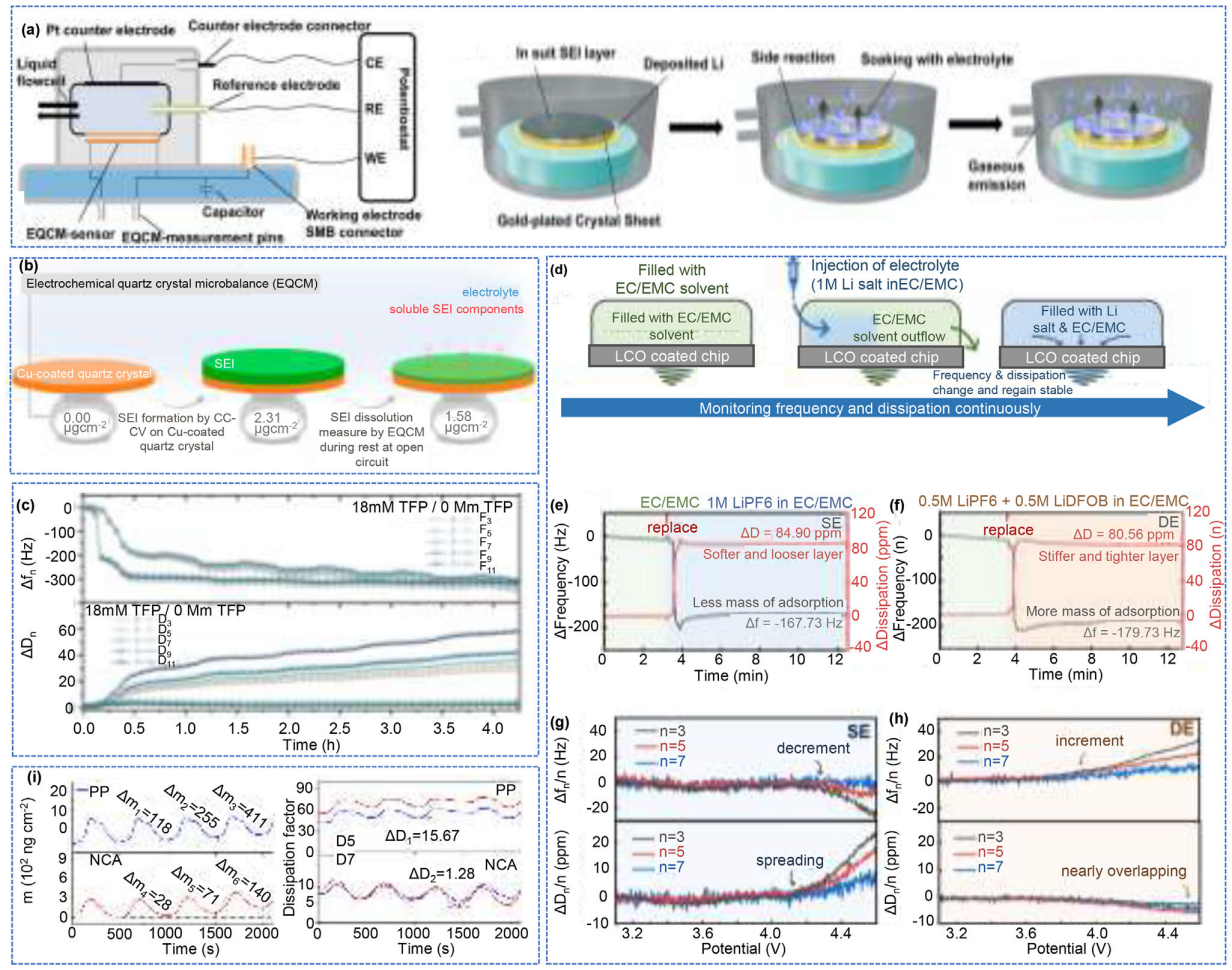
(a) EQCM working principle, measurement process schematic, and time evolution of the quartz crystal resonance frequency during operation, with the corresponding lithium mass change (∆*m*) shown in the inset. Reproduced with permission [140]. Copyright 2025, John Wiley and Sons. (b) Process of SEI formation and dissolution using electrochemical quartz crystal microbalance (EQCM). Reproduced with permission [142]. Copyright 2023, American Chemical Society. (c) Frequency and dissipation change with time in DME + 1 m LiFSI and DME + 1 m LiFSI + 18 mm TFP electrolytes. Reproduced under the terms of the Creative Commons CC‐BY 3.0 license [143]. Copyright 2024, Royal Society of Chemistry. (d) Schematic of EQCM measuring the effect of anion adsorption and solvation changes on the LCO interface; (e,f) Frequency and dissipation changes before and after replacing EC/EMC solvents with SE and DE (*n* = 7) for LCO‐coated chips. Reproduced with permission [145]. Copyright 2025, John Wiley and Sons. (g,h) Frequency and dissipation changes during charging in SE and DE electrolytes [145]. (i) In situ EQCM measurement of mass changes over time in Na||Cu batteries and ΔD at different harmonics. Reproduced with permission [146]. Copyright 2025, John Wiley and Sons.

SEI is a dynamically evolving system during cycling, with the formation, dissolution, regeneration, and mechanical degradation processes being intertwined. Wang et al. used EQCM to quantify the formation of irreversible SEI during Li deposition, with the test process and results shown in Figure 13a. The two tested systems, TTD with undiluted electrolyte and DCL with diluted electrolyte, directly confirmed that the semi‐solvated hexafluoroisopropyl methyl ether (HFME) diluent, through its lithium‐affinitive group, cooperatively coordinates with Li^+^, optimizing its desolvation kinetics and promoting the formation of a high‐quality SEI film rich in inorganic components [140]. Berg et al. used operando multi‐harmonic EQCM‐D together with EIS to study SEI formation on carbon and Cu electrodes in Li‐ion batteries. They found that electrolyte reduction products diffused through the electrolyte and deposited on the electrode surface, forming viscoelastic SEI layers. On Cu electrodes, a rigid LiF‐rich interphase formed above 1.5 V, while further electrolyte reduction below 0.8 V generated a softer polymer‐rich outer layer [141]. Kitz et al. employed operando multi‐harmonic EQCM‐D combined with EIS to investigate SEI formation on carbon anodes in Li‐ion batteries with vinylene carbonate(VC)‐ and fluoroethylene carbonate(FEC)‐containing electrolytes. EQCM‐D revealed that both additives reduced the overall SEI mass, while VC formed a thinner and more stable interphase with better passivation behavior. In contrast, FEC led to a more rigid but continuously growing SEI layer. By combining dissipation analysis with viscoelastic modeling, the study further quantified the mechanical properties and transport behavior of the SEI during cycling [45]. Philaphon et al. used EQCM to track and quantify the solubility of SEI components during the cycling of lithium metal anodes (LMA), as shown in Figure 13b. Their study found that the dissolution of SEI is a continuous dynamic process that leads to irreversible mass loss. By comparing different electrolyte systems, EQCM results directly established a quantitative relationship between SEI solubility, passivation performance, Coulombic efficiency, and cycling stability of LMA. The conclusion pointed out that the ongoing dissolution of SEI is the major contributing mechanism to capacity degradation and performance variations in LMA during cycling. This finding emphasizes that in electrolyte design, inhibiting SEI dissolution is crucial for achieving high‐stability, long‐cycle‐life lithium metal batteries [142]. Chulgi et al. proposed a method for preparing SEI films through the electrostatic attraction between fluoride cations and negatively charged anodes. In electrolytes with fluoride additives, EQCM‐D testing showed that the frequency change (Δ*f*/*n*) and energy dissipation (Δ*D*/*n*) were independent of the harmonic number (*n*), as shown in Figure 13c. This indicates that the formed interface is rigid, and after the first cycle, no further mass increase occurred. The fluorine‐rich interface generated on the surface exhibited excellent passivation effects, effectively blocking further solvent decomposition. In contrast, the electrolyte without additives showed continuous increases in mass and dissipation, indicating the formation of a loose and porous interface, which is the main cause of high overpotential and failure of the battery [143]. Berg et al. combined operando EQCM‐D with OEMS to study the effect of water contamination on SEI formation on carbon anodes. They found that adding 2000 ppm H_2_O increased the SEI thickness from ∼85 to ∼173 nm and promoted the formation of a more rigid Li_2_CO_3_‐rich interphase. EQCM‐D further revealed dynamic changes in SEI mass, stiffness, and electrolyte viscosity during cycling, indicating continued water reduction and interphase evolution at low potentials [144].

The properties of the CEI largely depend on the ionic adsorption behavior on the Helmholtz plane during the initial stages. This microscopic‐level interface reconstruction often dictates the stability of the subsequent passivation film. Ji et al. used EQCM‐D to directly observe the impact of anion adsorption on the Helmholtz plane on CEI formation using LiCoO_2_(LCO) as an example, as shown in Figure 13d. The baseline electrolyte was 1 m LiPF_6_ dissolved in EC and EMC at a volumetric ratio of 3:7 (denoted as SE), and LiDFOB replaced an equimolar amount of LiPF_6_ (denoted as DE). As shown in Figure 13e,f, due to the stronger adsorption tendency of DFOB^−^ compared to PF_6_^−^, the frequency of DE dropped more significantly, but its Δ*D* value changed less, possibly due to its rigid adsorption layer and lower coupling tendency with the solution medium. Monitoring the charging process reveals the simultaneous occurrence of two distinct processes: delithiation (Δ*m *< 0) and the formation of the CEI (Δ*m* > 0), as illustrated in Figure 13g,h. In SE, the continuous increase in mass indicated that carbonate molecules continued to decompose, while the increasing D value suggested that the resulting CEI was softer and richer in organic materials. In DE, the mass loss from delithiation was much greater than the mass accumulated from electrolyte decomposition and CEI formation, indicating that CEI in DE effectively prevented the continued decomposition of solvents. The *D* value in DE decreased across all three harmonics and overlapped, suggesting a uniform, rigid, and inorganic‐rich CEI [145]. Sel et al. developed a new “embedded piezoelectric sensor battery design” that enables galvanostatic charge–discharge measurements at low C‐rates (e.g., C/10 and C/20), thereby avoiding the issue that nanoampere‐level currents from low‐mass‐loaded QCM electrodes can be overwhelmed by parasitic circuit currents. In the LiFePO_4_//Graphite system, operando EQCM measurements revealed irreversible mass accumulation during the initial cycle, which was attributed to CEI formation with an estimated average thickness of ≈2.7 nm. In the NVPF//HC system, the measured mass evolution originated from the competition between desodiation‐induced mass loss and mass uptake associated with electrolyte decomposition and CEI formation. At lower C‐rates, electrolyte degradation contributed more significantly to mass accumulation, whereas at higher C‐rates the mass variation was dominated by desodiation, since electrolyte decomposition became kinetically limited [147]. Li et al. used EQCM to quantitatively assess the CEI formation process, mass change patterns, and mechanical properties of the Na_0.72_Ni_0.32_Mn_0.68_O_2_(NNM) cathode surface. In the baseline electrolyte, the Δ*D* fluctuated significantly (−2 to 4) and showed continuous irreversible mass increases, indicating that the formed CEI structure was loose and evolved continuously during cycling, undergoing repeated dissolution and regeneration. When a locking‐chain sodium 4,4′‐(1,4‐phenylenebis(oxy))‐bis(butane‐1‐sulfonate)‐15‐crown‐5 (15PBS) additive was added, the viscoelasticity remained stable, indicating that the CEI formed by 15PBS could effectively accommodate the volume changes during sodium ion extraction/insertion, and the NNM cathode achieved up to 93% average reversible mass ratio [148].

EQCM‐D can precisely distinguish reversible metal deposition/stripping from irreversible side reactions (such as electrolyte decomposition, SEI, and transition metal ion deposition), making it a powerful tool for revealing the failure mechanisms of metal anodes, evaluating the effects of separator or electrolyte additives, and guiding interface stabilization designs. Zhang et al. used EQCM to study the effects of transition metal ions (TMI) on the formation of SEI on graphite anodes. EQCM accurately distinguished and quantified the irreversible reduction and deposition of TMIs on the graphite electrode surface, leading to mass increases. The results showed that TMI deposition significantly altered the subsequent SEI formation process, with TMI deposits disrupting the normal SEI structure, leading to thicker and more unstable SEI films, which exhibited higher rates of sustained mass growth during cycling, directly related to the decrease in coulombic efficiency [149]. Guo et al. designed a polar nylon 6‐cellulose acetate (NCA) separator for Na/Mg batteries. The mass loss during the first three cycles with the NCA separator was much lower than that with the PP separator, as shown in Figure 13i, suggesting that the NCA separator facilitated the reversible sodium deposition and stripping process. The mechanical properties of the generated SEI were assessed by fluctuations in the dissipation factor, as shown in Figure 13i. The SEI formed with the NCA separator was more stable, forming a uniformly distributed organic/inorganic viscoelastic SEI structure that alleviated volume expansion and electron accumulation, significantly inhibiting Na dendrite growth. In contrast, the PP separator showed higher organic content, leading to dissolution and regeneration behaviors (Δ*D* = 15.67), which inevitably caused Na^+^ accumulation and dendrite growth, thus reducing battery performance [146]. Krumov et al. used EQCM to directly track the dynamic evolution of SEI on lithium metal anodes, accurately distinguishing and quantifying the irreversible mass increase caused by electrolyte decomposition and SEI formation during lithium deposition/stripping, as well as the mass loss due to SEI dissolution or mechanical degradation. They revealed the stable passivation formation of SEI at specific potentials (manifested as a decrease in mass growth rate) and the continued fluctuation of mass during subsequent cycles, which directly reflected the dynamic stability and degradation tendency of the SEI structure [150]. Saida et al. used EQCM‐D to deeply study the dynamic formation process of SEI on the zinc electrode in both aqueous and non‐aqueous electrolytes, as well as the reversible Zn deposition/dissolution behavior. By comparing actual mass changes with theoretical reversible Zn deposition/dissolution mass, they quantified the irreversible mass loss caused by continuous SEI growth or side reactions (such as hydrogen evolution) during each cycle, directly reflecting the cycling stability and deposition efficiency of the Zn electrode [151].

### 3.4 EQCM Combined With Other Techniques

Due to inherent limitations, EQCM can only reflect mass balance and cannot identify the sources of mass flux in complex systems. Generally, when the mass change measured during the experiment exceeds the mass of bare ions calculated from Faraday's law, it suggests that solvent molecules or co‐ions may be involved in the electrochemical process. However, EQCM‐related models are unable to differentiate between the electrochemical processes of solvated ions and co‐ions. In such cases, appropriate complementary approaches can be employed for joint analysis. Computational methods include molecular dynamics (MD) simulations and density functional theory [91, 152]. Experimental characterization techniques include X‐ray diffraction (XRD) [153, 154], Raman spectroscopy [155, 156], Fourier transform infrared spectroscopy (FTIR) [157, 158], Nuclear magnetic resonance (NMR) [129, 159], and scanning transmission X‐ray microscopy (STXM) [160, 161], as well as additional techniques such as X‐ray photoelectron spectroscopy (XPS) [162, 163], ellipsometry [164, 165], electrochemical mass spectrometry (OEMS/DEMS) [144, 166], and atomic force microscopy [167, 168], thereby enabling more comprehensive and multidimensional characterization of interfacial processes.

#### 3.4.1 Combining EQCM With Other Experimental Methods

**FIGURE 14:**
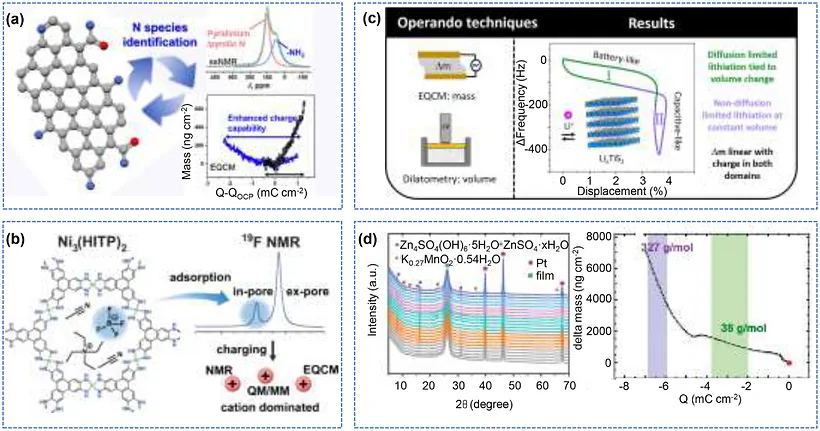
(a) Illustration of the study of capacitive charge storage mechanism in nitrogen‐doped porous carbon electrodes using ssNMR and EQCM. Reproduced with permission [170]. Copyright 2022, American Chemical Society. (b) Study of ion adsorption and charging mechanism in Ni_3_(HITP)_2_ supercapacitors using NMR and EQCM. Reproduced under the terms of the Creative Commons CC‐BY 4.0 license [171]. Copyright 2024, American Chemical Society. (c) Illustration of the two‐phase lithiation process in TiS_2_ using EQCM combined with EDC. Reproduced with permission [173]. Copyright 2024, Elsevier. (d) In situ XRD spectra of K_0.27_MnO_2_·0.54H_2_O in 2 m ZnSO_4 _+ 0.3 m MnSO_4_ electrolyte during reduced polarization showing the relationship between electrode mass change and charge. Reproduced under the terms of the Creative Commons CC‐BY‐NC‐ND license [174]. Copyright 2021, John Wiley and Sons.

The combination of in situ NMR with EQCM provides a powerful analytical tool for studying the microscopic mechanisms and macroscopic behaviors of the EEI. NMR can qualitatively observe changes in the local environment of ions within pores, while EQCM can quantitatively measure changes in the total mass of the electrode. The combined technique can track the displacement of all types of electrolyte species, including cations, anions, and solvent molecules, thereby providing a comprehensive description of the interface structure [169]. Kaskel and co‐workers combined EQCM with low‐temperature solid‐state NMR to deeply analyze the capacitive charge storage mechanism of nitrogen‐doped porous carbon electrodes, as shown in Figure 14a. By correlating the chemical structure (ssNMR) with mass transport behavior (EQCM), this study demonstrated, at the molecular level, that nitrogen doping induces a unique synergistic adsorption mechanism for cations and anions [170]. Forse and co‐workers used external‐field NMR and in situ EQCM experiments to study the charge storage mechanism in Ni_3_(HITP)_2_ MOF supercapacitors, revealing that cations are the main contributors to charge storage, while anions contribute less, as illustrated in Figure 14b. Solid‐state NMR revealed different resonance peaks for ions inside and outside the pores, showing that the chemical shift of the ions inside the pores is primarily dominated by specific interactions between the MOF and the electrolyte [171]. Huang et al. used in situ EQCM and NMR to clarify the co‐intercalation process of solvated Na^+^‐diglyme ([Na‐*x*G_2_]^+^) in graphite and its electrochemical behavior. Importantly, the authors explicitly evaluated the dissipation factor during EQCM measurements; the small variation in dissipation, Δ*D* = 0.04–0.5 × 10^−6^, indicated the formation of a relatively rigid electrode film and supported the applicability of the Sauerbrey equation for calculating mass changes. Based on this gravimetric condition, EQCM monitored the dynamic intercalation of Na^+^ and G_2_ solvent, revealing the changes in the ratio of Na^+^ to G_2_ solvent during intercalation. NMR provided insights into the coordination environment of Na^+^ with the graphite layers at different stages of the intercalation process. The combination of these two techniques provided key molecular‐level insights into the electrochemical behavior of the [Na‐xG_2_]^+^ co‐intercalation process and the spatial evolution of solvated ions within the graphite layers [172]. This combined methodology of monitoring mass changes and ion species quantitatively is crucial for understanding the structure–performance relationships and the desolvation dynamics at the EEI.

The electrochemical dilatometer (ECD) can provide real‐time information about the expansion and contraction of electrodes during the electrochemical process. The combination of ECD and EQCM establishes a ternary correlation among mass change, volume expansion, and potential during the charge storage process. This synergistic approach provides irrefutable experimental evidence for the precise deconvolution of complex processes characterized by disparate diffusion kinetics and interlayer parameters. Furthermore, these insights offer critical guidance for the rational design and optimization of electrode materials to achieve both superior capacity and high‐rate performance. As shown in Figure 14c, Simon et al. employed a hyphenated EQCM and ECD strategy to unambiguously differentiate two discrete stages during the lithiation process of TiS_2_. In the first stage (diffusion‐limited), the initial lithiation regime starting from Li_0_TiS_2_ exhibits battery‐like behavior, where EQCM‐captured mass changes correlate with significant volume expansion recorded by ECD, signifying a rapid increase in interlayer spacing upon Li^+^ intercalation. The second stage (non‐diffusion‐limited) occurs once the interlayer spacing reaches a maximum, and subsequent lithiation is non‐diffusion‐limited (capacitive‐like behavior), where the mass change tracked by EQCM is small, suggesting that the mechanism shifts from a structure‐driven phase change to surface/interface adsorption or intercalation [173]. Mennatalla et al. used EQCM and ECD to investigate the effect of interlayer spacing changes in hydrogen titanates on charge storage mechanisms. They found that after supporting with organic molecular pillars, the interlayer expansion led to a shift in the charge storage mechanism from desolvated Li^+^ intercalation to ion‐solvent co‐intercalation. ECD confirmed that the non‐crosslinked pillar‐supported structure exhibited better mechanical flexibility and could accommodate intercalation‐induced expansion [154]. Ge et al. studied the influence of cation–electrode interactions on electrochemical behavior using rGO as a model material through EQCM and ECD. Their combined technique revealed that charge storage occurs in two stages. In Region I, cations intercalate into the rGO interlayers in a solvated form, and ECD monitors the increase in electrode thickness. In Region II, at higher cathodic polarization, cations intercalate as bare ions or partially desolvated ions, depending on the strength of the interaction between the cations and the rGO surface. The stronger the interaction, the higher the degree of desolvation and the greater the capacity. A significant decrease in the rate of electrode thickness increase was also observed, revealing that desolvation enhances the capacitance [175]. This hyphenated technique transcends the inherent limitations of standalone methodologies, enabling real‐time, synchronized tracking of gravimetric and volumetric evolutions. It establishes a robust coupling between microscopic ionic flux behaviors and macroscopic structural strain effects.

In situ spectroscopic methods can identify and track the voltage‐dependent changes of molecular species and chemical bonds at the EEI in real time. However, these methods cannot monitor the internal pores of electrodes, where EQCM‐D excels in evaluating ion transport mechanisms, solvation effects, and macroscopic structural characteristics of porous electrodes, effectively complementing spectroscopic techniques' limitations in monitoring internal electrode behavior. This enables a comprehensive analysis of the dynamic processes at complex solid–liquid interfaces, including chemical composition, mass conservation, and mechanical properties. For example, combining ATR‐SEIRAS with EQCM‐D confirmed that different electrolyte anions in zinc‐ion batteries change the hydration structure of Zn^2+^, thereby affecting its dehydration potential barrier and deposition behavior at the electrode surface. Specifically, the addition of ACN to the high‐concentration acetonitrile‐water‐in‐salt electrolyte successfully modulated the mass and properties of the interfacial film by affecting the solvation shell of Zn^2+^, which helped suppress side reactions like hydrogen evolution [176]. In the zinc storage study using polyaniline (PANI), EQCM‐Raman synchronicity confirmed the limited intercalation of Zn^2+^ and structural rearrangement of the PANI framework [177]. Liu et al. combined in situ XRD (shown in Figure 14c) and EQCM to confirm that ZHS precipitation and H_3_O^+^ intercalation occur simultaneously, revealing a two‐step reduction mechanism: H_3_O^+^ intercalation at 1.4 V (∼38 g/mol) and ZHS precipitation at 1.2 V (∼327 g/mol), as shown in Figure 14d. EQCM provides direct gravimetric evidence for the coupled mechanisms of ion‐selective intercalation and chemical precipitation, effectively compensating for the insensitivity of XRD toward light elements (e.g., hydrogen) [174]. Wang et al. used EQCM‐D and XPS to study the formation and stability of SEI on the zinc anode in a DG‐containing electrolyte. They found that the coordinated DG and OTF^−^ underwent reduction and decomposition to form a self‐healing SEI composed of inorganic/organic ZnF_2_‐ZnS, effectively suppressing harmful side reactions and guiding uniform Zn deposition [178]. Simon et al. combined EQCM with various in situ characterization techniques to elucidate the different physical properties during cation intercalation/de‐intercalation in Ti_3_C_2_T*_x_* in WIS and SIW LiCl electrolytes, emphasizing that the electrochemical behavior of active materials is influenced not only by their inherent properties but also by their interactions with the electrolyte [179]. These studies demonstrate the unique advantages of combining in situ characterization techniques in the field of electrochemical energy storage. By providing comprehensive and high‐precision dynamic monitoring of EEI processes, these hyphenated approaches offer invaluable experimental foundations for the rational design, optimization, and interfacial engineering of advanced electrode materials.

#### 3.4.2 Integration of EQCM and Calculation Methods

**FIGURE 15:**
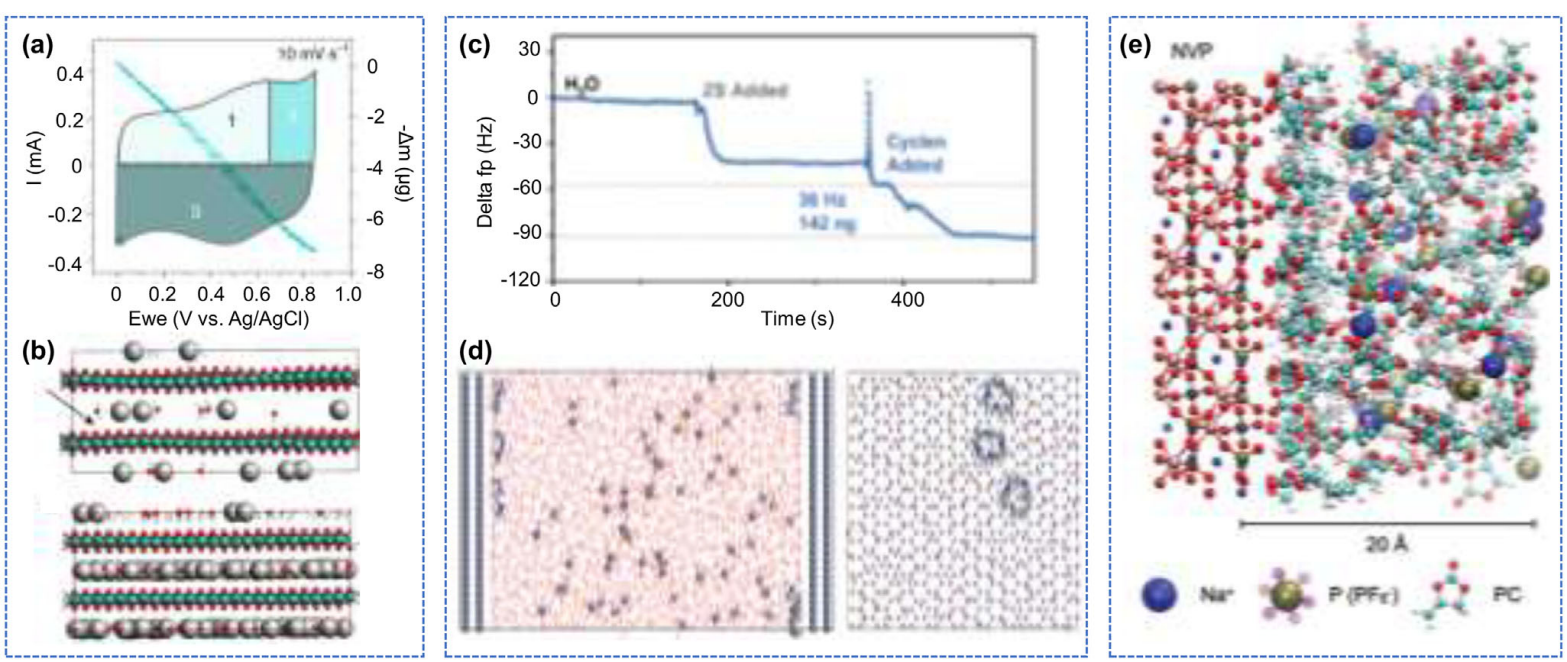
(a) Relationship between mass change and potential of birnessite in K_2_SO_4_ electrolyte; (b) ReaxFF GCMC simulation snapshots showing the intercalation of K^+^ and H_2_O into birnessite: O (red), H (light gray), K^+^ (dark gray), and Mn (cyan) [152]. (c) EQCM curves after the addition of different species; (d) Species distribution obtained from MD simulations [180]. (e) MD simulations of the NVP‐PC system at equilibrium without applied voltage [181].

By combining real‐time EQCM monitoring of mass changes with MD simulations for microscopic dynamical analysis, researchers can gain deep insights into complex phenomena occurring during charge storage processes. For example, the distinction between electric double layer capacitance and pseudocapacitance in nanostructured electrodes has long been a subject of debate. Shelby et al. conducted a detailed study on the capacitive charge storage mechanism of birnessite by integrating ex situ XRD, EQCM, and MD simulation (shown in Figure 15a,b). They found that cation insertion and extraction dominate the charge storage process, and that most insertion‐dominated processes do not exhibit capacitive behavior. MD simulations reveal that the capacitive characteristics of interfacial intercalation originate from the presence of nanoconfined interlayer structural water. This hydration layer maintains a significant distance between the intercalated cations and the Mn coordination centers, thereby minimizing structural distortion and lattice evolution during the charging/discharging process [152]. As shown in Figure 15c,d, by combining real‐time EQCM with constant‐potential MD simulations, Duan et al. demonstrated that the dynamic adsorption behavior in response to an alternating electric field is pivotal for modulating the metal–electrolyte interface. From a microscopic perspective, they revealed the dynamic characteristics of the zinc/electrolyte interface during repeated zinc plating/stripping and clarified the optimization mechanisms of electrolyte additives at this interface. Furthermore, this molecular‐level understanding provides substantive guidance for the selection and optimization of such additives [180]. Ezzoubair et al. adopted a combined approach of EQCM, operando infrared fiber evanescent wave spectroscopy (IR FEWS), and MD simulations to investigate the electrode/electrolyte interface in Na_3_V_2_(PO_4_)_3_(NVP) and Na_3_V_2_(PO_4_)_2_F_3_(NVPF) electrodes. At the NVP interface, anomalous mass uptake/consumption signals were observed, corresponding to the desolvation process of Na^+^ insertion into the NVP framework, and this interpretation was corroborated by MD simulations (shown in Figure 15e). The desolvation process creates opposing solvent and bare Na^+^ concentration gradients, causing solvent molecules to migrate away from the detection boundary of the EQCM resonator. In contrast, for NVPF, whose bulk diffusion kinetics are relatively slower, no mass loss peaks associated with desolvation were observed [181]. Overall, EQCM and its coupling with complementary techniques have become critical tools for revealing ion transport behaviors at energy storage material interfaces and for optimizing electrode/electrolyte matching.

## 4 Conclusion and Outlook

This review systematically summarizes recent advances in the application of EQCM to supercapacitors and battery systems. Benefiting from its exceptional sensitivity and real‐time capability in monitoring mass variations and energy dissipation factors, EQCM has evolved into a central diagnostic platform for investigating EEI. Its pivotal role is demonstrated across several critical dimensions: (1) elucidating charge storage mechanisms and ion transport kinetics; (2) guiding interfacial engineering and the rational design of electrodes/electrolytes; (3) evaluating structural evolution and failure/degradation pathways of electrodes; (4) monitoring parasitic interfacial reactions and the formation of interphases; and (5) enabling multi‐dimensional analysis through integration with complementary techniques. The fundamental insights gained from EQCM‐based interfacial studies not only deepen our understanding of the underlying physics of energy storage but also provide transformative design principles for next‐generation high‐performance energy storage devices.

Despite these significant advancements, several critical challenges remain to be addressed to fully exploit the potential of EQCM in developing advanced energy storage systems: (1) Deep integration of multimodal in situ techniques. Future efforts should prioritize the complementary use of EQCM with in situ spectroscopy (e.g., Raman, FTIR), NMR, electrochemical microscopy, and XPS to achieve correlated analysis of chemical composition, structural evolution, mass transport, and mechanical properties. Such an integrated approach is essential for elucidating interfacial dynamics and the interplay between chemical flux and mechanical stability. (2) Extension to complex systems and extreme conditions. The application scope of EQCM should be expanded to high‐concentration electrolytes, solid/quasi‐solid‐state electrolytes, and multi‐field coupling environments (e.g., extreme temperatures). However, for highly viscous or mechanically complex systems, particular attention should be paid to acoustic‐wave damping and non‐rigid loading effects, which may complicate the interpretation of frequency and dissipation signals. Therefore, more rigorous physical compensation models are required for complex porous electrodes and flexible materials to improve the reliability of quantitative gravimetric analysis.

(3) Synergy with computational science and intelligent data analytics. Integration of MD, DFT, and machine learning can substantially enhance signal decoupling and mechanism identification, while establishing a structure‐property relationship linking microscopic interfacial parameters to macroscopic device performance (e.g., energy density, power density, and cycling life) will enable the rational design of high‐performance electrode materials and interfaces, and accelerate the development cycle.

In summary, EQCM is evolving from a single mass‐monitoring technique into a multi‐method, integrated characterization platform. In future energy storage research, EQCM will not only be employed to measure mass variations but will also play a central role in quantitatively elucidating the dynamic evolution of EEI through integration with diverse in situ techniques and continued advances in data analysis. Such in‐depth mechanistic understanding will facilitate the optimization of existing supercapacitor and battery systems and provide critical experimental support for the development of emerging energy storage technologies, including all‐solid‐state batteries and multivalent‐ion batteries. By establishing accurate structure–property relationship models, EQCM can directly enable efficient materials screening and rational interface engineering. This precise characterization based on in situ mass monitoring will serve as an important bridge between microscopic reaction mechanisms and macroscopic device performance, fundamentally promoting the development of high‐efficiency energy storage materials and the sustained improvement of device performance.

## Author Contributions

**Zixuan Wang**: Writing – original draft, investigation, visualization, data curation. **Situo Cheng**: writing – original draft, writing – review and editing, supervision, funding acquisition, investigation, conceptualization, validation. **Guang Feng**: writing – review and editing, resources, supervision, project administration, investigation, conceptualization, methodology.

## Conflicts of Interest

The authors declare no conflicts of interest.

## Data Availability

The data that support the findings of this study are available from the corresponding author upon reasonable request.

## References

[1] G. Zhou , L. Xu , G. Hu , L. Mai , and Y. Cui , “Nanowires for Electrochemical Energy Storage,” Chemical Reviews 119 (2019): 11042–11109, 10.1021/acs.chemrev.9b00326. 31566351

[2] M. Chen , Y. Zhang , G. Xing , S.‐L. Chou , and Y. Tang , “Electrochemical Energy Storage Devices Working in Extreme Conditions,” Energy & Environmental Science 14 (2021): 3323–3351, 10.1039/d1ee00271f.

[3] X. Wang , M. Salari , D.‐E. Jiang , et al., “Electrode Material–Ionic Liquid Coupling for Electrochemical Energy Storage,” Nature Reviews Materials 5 (2020): 787–808, 10.1038/s41578-020-0218-9.

[4] S. Wei , S. Choudhury , Z. Tu , et al., “Electrochemical Interphases for High‐Energy Storage Using Reactive Metal Anodes,” Accounts of Chemical Research 51, no. 1 (2017): 80–88, 10.1021/acs.accounts.7b00484. 29227617

[5] J. Hui , Z. T. Gossage , D. Sarbapalli , et al., “Advanced Electrochemical Analysis for Energy Storage Interfaces,” Analytical Chemistry 91, no. 1 (2018): 60–83, 10.1021/acs.analchem.8b05115. 30428255

[6] A. Mishra , D. Sarbapalli , O. Rodríguez , and J. Rodríguez‐López , “Electrochemical Imaging of Interfaces in Energy Storage via Scanning Probe Methods: Techniques, Applications, and Prospects,” Annual Review of Analytical Chemistry 16 (2023): 93–115, 10.1146/annurev-anchem-091422-110703. 37068746

[7] A. Patra , N. K , and J. R. Jose , “Understanding the Charge Storage Mechanism of Supercapacitors: in Situ/Operando Spectroscopic Approaches and Theoretical Investigations,” Journal of Materials Chemistry A 9, no. 46 (2021): 25852–25891, 10.1039/D1TA07401F.

[8] H. Wu , S. J. Zhang , Y. Jiang , et al., “Self‐Discharge Behaviors in Aqueous Batteries,” Angewandte Chemie International Edition 65 (2025): 20601, 10.1002/anie.202520601. 41472448

[9] T. Liu , L. Lin , X. Bi , et al., “In Situ Quantification of Interphasial Chemistry in Li‐Ion Battery,” Nature Nanotechnology 14, no. 1 (2018): 50–56, 10.1038/s41565-018-0284-y. 30420761

[10] W. Gao , N. Krins , C. Laberty‐Robert , H. Perrot , and O. Sel , “Scrutiny of the LiCoO_2_ Composite Electrode/Electrolyte Interface by Advanced Electrogravimetry and Implications for Aqueous Li‐Ion Batteries,” Journal of Physical Chemistry C 125 (2021): 3859–3867, 10.1021/acs.jpcc.0c09708.

[11] A. O. Efremova , A. I. Volkov , E. G. Tolstopyatova , and V. V. Kondratiev , “EQCM Study of Intercalation Processes into Electrodeposited MnO_2_ Electrode in Aqueous Zinc‐Ion Battery Electrolyte,” Journal of Alloys and Compounds 892 (2022): 162142, 10.1016/j.jallcom.2021.162142.

[12] F. Ye , Q. Liu , H. Dong , et al., “Organic Zinc‐Ion Battery: Planar, π‐Conjugated Quinone‐Based Polymer Endows Ultrafast Ion Diffusion Kinetics,” Angewandte Chemie International Edition 61, no. 51 (2022): 202214244, 10.1002/anie.202214244. 36285465

[13] J. L. S. Antonio , V. L. Martins , S. I. Córdoba de Torresi , and R. M. Torresi , “QCM‐D Study of Electrochemical Synthesis of 3D Polypyrrole Thin Films for Negative Electrodes in Supercapacitors,” Electrochimica Acta 324 (2019): 134887, 10.1016/j.electacta.2019.134887.

[14] A. C. Forse , C. Merlet , J. M. Griffin , and C. P. Grey , “New Perspectives on the Charging Mechanisms of Supercapacitors,” Journal of the American Chemical Society 138 (2016): 5731–5744, 10.1021/jacs.6b02115. 27031622 PMC4865825

[15] S. Fan , Z. Yan , D. Tang , et al., “Revealing Capacitive Slope Capacity of Open Pore Carbon for Ultrahigh‐Rate Sodium‐Ion Storage,” Advanced Materials 38, no. 3 (2025): 13223, 10.1002/adma.202513223. 41041967

[16] J. Wang , B. Tian , Y. Cheng , et al., “Polyacrylonitrile Alcoholysis and Freeze‐Expanded Assistive Synthesis of Porous Carbon for High Rate Supercapacitors and Zinc‐Ion Hybrid Capacitors,” Chemsuschem 18, no. 15 (2025): 202500787, 10.1002/cssc.202500787. 40476356

[17] S. Slesinska , P. Galek , J. Menzel , S. W. Donne , K. Fic , and A. Platek‐Mielczarek , “Fundamentals and Implication of Point of Zero Charge (PZC) Determination for Activated Carbons in Aqueous Electrolytes,” Advanced Science 11, no. 48 (2024): 2409162, 10.1002/advs.202409162. 39535367 PMC11672325

[18] H. Zhang , H. Wang , W. Li , et al., “Enabling High‐Performance Potassium‐Ion Batteries by Manipulating Interfacial Chemistry,” Advanced Functional Materials 34, no. 21 (2024): 2312368, 10.1002/adfm.202312368.

[19] S. Cheng , Z. Cao , Y. Liu , et al., “Ballistic Electrolyte Ion Transport with Undisturbed Pathways for Ultrahigh‐Rate Electrochemical Energy Storage Devices,” Energy & Environmental Science 17 (2024): 1997–2006, 10.1039/D3EE03819J.

[20] W.‐Y. Tsai , P.‐L. Taberna , and P. Simon , “Electrochemical Quartz Crystal Microbalance (EQCM) Study of Ion Dynamics in Nanoporous Carbons,” Journal of the American Chemical Society 136 (2014): 8722–8728, 10.1021/ja503449w. 24869895

[21] M. D. Levi , N. Levy , S. Sigalov , G. Salitra , D. Aurbach , and J. Maier , “Electrochemical Quartz Crystal Microbalance (EQCM) Studies of Ions and Solvents Insertion into Highly Porous Activated Carbons,” Journal of the American Chemical Society 132 (2010): 13220–13222, 10.1021/ja104391g. 20828121

[22] X. Yang , L. Wu , L. Ye , et al., “Bioinspired High‐Rate Supercapacitor Diode for Kilohertz Computing‐in‐Memory Application,” Advanced Functional Materials 36, no. 17 (2025): 24006, 10.1002/adfm.202524006.

[23] W. Kim , T. H. Kim , J. Yu , Y.‐J. Kim , K. J. Kim , and H.‐S. Kim , “Interface‐Targeting Individually Functionalized Ionic Additive to Construct Stable Interphase on Selective Electrode Surface for Practical Lithium‐Ion Pouch Cells,” Advanced Functional Materials 33, no. 49 (2023): 2306068, 10.1002/adfm.202306068.

[24] D. Aurbach and A. Zaban , “The Application of EQCM to the Study of the Electrochemical Behavior of Propylene Carbonate Solutions,” Journal of Electroanalytical Chemistry 393 (1995): 43–53, 10.1016/0022-0728(95)04014-F.

[25] D. A. Buttry and M. D. Ward , “Measurement of Interfacial Processes at Electrode Surfaces with the Electrochemical Quartz Crystal Microbalance,” Chemical Reviews 92 (1992): 1355–1379, 10.1021/cr00014a006.

[26] M. D. Ward and D. A. Buttry , “Situ Interfacial Mass Detection with Piezoelectric Transducers,” Science 249, no. 4972 (1990): 1000, 10.1126/science.249.4972.1000. 17789608

[27] V. Vanoppen , D. Johannsmann , X. Hou , J. Sjölund , P. Broqvist , and E. J. Berg , “Exploring Metal Electroplating for Energy Storage by Quartz Crystal Microbalance: a Review,” Advanced Sensor Research 3, no. 9 (2024): 2400025, 10.1002/adsr.202400025.

[28] N. Kornienko , “Feeling the Weight,” Nature Catalysis 5 (2022): 852–853, 10.1038/s41929-022-00860-w.

[29] H. L. Bandey , S. J. Martin , R. W. Cernosek , and A. R. Hillman , “Modeling the Responses of Thickness‐Shear Mode Resonators under Various Loading Conditions,” Analytical Chemistry 71 (1999): 2205–2214, 10.1021/ac981272b. 21662758

[30] M. Cassiède , J. H. Paillol , J. Pauly , et al., “Electrical Behaviour of AT‐Cut Quartz Crystal Resonators as a Function of Overtone Number,” Sensors And Actuators A: Physical 159, no. 2 (2010): 174–183, 10.1016/j.sna.2010.03.028.

[31] J. Curie and P. Curie , “Développement Par Compression De L'électricité Polaire dans les Cristaux Hémièdres à Faces Inclinées,” Bulletin de la Société minéralogique de France 3, no. 4 (1880): 90–93, 10.3406/bulmi.1880.1564.

[32] G. Sauerbrey , “Verwendung Von Schwingquarzen Zur Wägung Dünner Schichten und Zur Mikrowägung,” Zeitschrift für Physik 155, no. 2 (1959): 206–222, 10.1007/BF01337937T.

[33] T. Nomura and M. Okuhara , “Frequency Shifts of Piezoelectric Quartz Crystals Immersed in Organic Liquids,” Analytica Chimica Acta 142 (1982): 281–284, 10.1016/S0003-2670(01)95290-0.

[34] C. Leppin , A. Peschel , F. S. Meyer , A. Langhoff , and D. Johannsmann , “Kinetics of Viscoelasticity in the Electric Double Layer Following Steps in the Electrode Potential Studied by a Fast Electrochemical Quartz Crystal Microbalance (EQCM),” Analyst 146 (2021): 2160–2171, 10.1039/d0an01965h. 33543737

[35] A. R. Hillman , “The EQCM: Electrogravimetry with a Light Touch,” Journal of Solid State Electrochemistry 15, no. 7–8 (2011): 1647–1660, 10.1007/s10008-011-1371-2.

[36] S. Bruckenstein and M. Shay , “Experimental Aspects of Use of the Quartz Crystal Microbalance in Solution,” Electrochimica Acta 30 (1985): 1295–1300, 10.1016/0013-4686(85)85005-2.

[37] K. K. Kanazawa and J. G. Gordon , “Frequency of a Quartz Microbalance in Contact with Liquid,” Analytical Chemistry 57, no. 8 (1985): 1770–1771, 10.1021/ac00285a062.

[38] S. Bourkane , C. Gabrielli , and M. Keddam , “Study of Electrochemical Phase Formation and Dissolution by Ac Quartz Electrogravimetry,” Electrochimica Acta 34 (1989): 1081–1092, 10.1016/0013-4686(89)87140-3.

[39] M. Rodahl , F. Hook , A. Krozer , P. Brzezinski , and B. Kasemo , “Quartz Crystal Microbalance Setup for Frequency and Q‐Factor Measurements in Gaseous and Liquid Environments,” Review of Scientific Instruments 66 (1995): 3924–3930, 10.1063/1.1145396.

[40] M. D. Levi , G. Salitra , N. Levy , et al., “Application of a Quartz‐Crystal Microbalance to Measure Ionic Fluxes in Microporous Carbons for Energy Storage,” Nature Materials 11, no. 8 (2009): 872–875, 10.1038/nmat2559. 19838184

[41] D. Johannsmann , A. Langhoff , and C. Leppin , “Studying Soft Interfaces with Shear Waves: Principles and Applications of the Quartz Crystal Microbalance (QCM),” Sensors 21 (2021): 3490, 10.3390/s21103490. 34067761 PMC8157064

[42] N. Shpigel , M. D. Levi , and D. Aurbach , “EQCM‐D Technique for Complex Mechanical Characterization of Energy Storage Electrodes: Background and Practical Guide,” Energy Storage Materials 21 (2019): 399–413, 10.1016/j.ensm.2019.05.026.

[43] N. Shpigel , M. D. Levi , S. Sigalov , L. Daikhin , and D. Aurbach , “In Situ Real‐Time Mechanical and Morphological Characterization of Electrodes for Electrochemical Energy Storage and Conversion by Electrochemical Quartz Crystal Microbalance with Dissipation Monitoring,” Accounts of Chemical Research 51 (2018): 69–79, 10.1021/acs.accounts.7b00477. 29297669

[44] S. N. Songkhla and T. Nakamoto , “Overview of Quartz Crystal Microbalance Behavior Analysis and Measurement,” Chemosensors 9 (2021): 350, 10.3390/chemosensors9120350.

[45] P. G. Kitz , M. J. Lacey , P. Novák , and E. J. Berg , “Operando Investigation of the Solid Electrolyte Interphase Mechanical and Transport Properties Formed from Vinylene Carbonate and Fluoroethylene Carbonate,” Journal of Power Sources 477 (2020): 228567, 10.1016/j.jpowsour.2020.228567.

[46] S. Liao , C. Ge , D. Qiu , et al., “BVD Model for QCM Loaded by Viscoelastic Film in Gas Phase Application,” AIP Advances 10, no. 7 (2020): 075119, 10.1063/5.0011532.

[47] I. Burda , “Assessing Impedance Analyzer Data Quality by Fractional Order Calculus: a Qcm Sensor Case Study,” Electronics 12 (2023): 2127, 10.3390/electronics12092127.

[48] M. D. Levi , L. Daikhin , D. Aurbach , and V. Presser , “Quartz Crystal Microbalance with Dissipation Monitoring (EQCM‐D) for in‐Situ Studies of Electrodes for Supercapacitors and Batteries: a Mini‐Review,” Electrochemistry Communications 67 (2016): 16–21, 10.1016/j.elecom.2016.03.006.

[49] M. D. Levi , N. Shpigel , S. Sigalov , et al., “In Situ Porous Structure Characterization of Electrodes for Energy Storage and Conversion by EQCM‐D: a Review,” Electrochimica Acta 232 (2017): 271–284, 10.1016/j.electacta.2017.02.149.

[50] K. Zheng , Y. Xian , and Z. Lin , “A Method for Deconvoluting and Quantifying the Real‐Time Species Fluxes and Ionic Currents Using in Situ Electrochemical Quartz Crystal Microbalance,” Advanced Materials Interfaces 9, no. 16 (2022): 2200112, 10.1002/admi.202200112.

[51] D. Benito , C. Gabrielli , J. J. Garci?a‐Jareño , M. Keddam , H. Perrot , and F. Vicente , “An Electrochemical Impedance and Ac‐Electrogravimetry Study of PNR Films in Aqueous Salt Media,” Electrochemistry Communications 4 (2002): 613–619, 10.1016/S1388-2481(02)00387-9.

[52] L. To Thi Kim , C. Debiemme‐Chouvy , and C. Gabrielli , “Redox Switching of Heteropolyanions Entrapped in Polypyrrole Films Investigated by Ac Electrogravimetry,” Langmuir 28, no. 38 (2012): 13746–13759, 10.1021/la302115f. 22931507

[53] J. Agrisuelas , C. Gabrielli , J. J. García‐Jareño , H. Perrot , and F. Vicente , “Effects of Anion Size on the Electrochemical Behavior of H_2_SO_4_‐Structured Poly(O‐Toluidine) Films. An Ac‐Electrogravimetry Study in Acid Solutions,” Electrochimica Acta 132 (2014): 561–573, 10.1016/j.electacta.2014.04.047.

[54] T. Lé , D. Aradilla , G. Bidan , et al., “Unveiling the Ionic Exchange Mechanisms in Vertically‐Oriented Graphene Nanosheet Supercapacitor Electrodes with Electrochemical Quartz Crystal Microbalance and Ac‐Electrogravimetry,” Electrochemistry Communications 93 (2018): 5–9, 10.1016/j.elecom.2018.05.024.

[55] C. Gabrielli , J. J. Garcia‐Jareño , M. Keddam , et al., “Ac‐Electrogravimetry Study of Electroactive Thin Films. Ii. Application to Polypyrrole,” Journal of Physical Chemistry B 106, no. 12 (2002): 3192–3201, 10.1021/jp013925p.

[56] C. Gabrielli , J. J. García‐Jareño , M. Keddam , et al., “Ac‐Electrogravimetry Study of Electroactive Thin Films. I. Application to Prussian Blue,” Journal of Physical Chemistry B 106, no. 12 (2002): 3182–3191, 10.1021/jp013924x.

[57] H. Goubaa , F. Escobar‐Teran , I. Ressam , et al., “Dynamic Resolution of Ion Transfer in Electrochemically Reduced Graphene Oxides Revealed by Electrogravimetric Impedance,” Journal of Physical Chemistry C 121 (2017): 9370–9380, 10.1021/acs.jpcc.7b01421.

[58] F. Escobar‐Teran , A. Arnau , J. V. Garcia , Y. Jiménez , H. Perrot , and O. Sel , “Gravimetric and Dynamic Deconvolution of Global EQCM Response of Carbon Nanotube Based Electrodes by Ac‐Electrogravimetry,” Electrochemistry Communications 70 (2016): 73–77, 10.1016/j.elecom.2016.07.005.

[59] C. R. Arias , C. Debiemme‐Chouvy , C. Gabrielli , et al., “New Insights into Pseudocapacitive Charge‐Storage Mechanisms in Li‐Birnessite Type MnO_2_ Monitored by Fast Quartz Crystal Microbalance Methods,” Journal of Physical Chemistry C 118 (2014): 26551–26559, 10.1021/jp508543h.

[60] E. Bendadesse , A. V. Morozov , A. M. Abakumov , H. Perrot , J.‐M. Tarascon , and O. Sel , “Deciphering the Double‐Layer Structure and Dynamics on a Model Li_x_MoO_3_ Interface by Advanced Electrogravimetric Analysis,” ACS Nano 16 (2022): 14907–14917, 10.1021/acsnano.2c05784. 35984450

[61] A. Bouzina , H. Perrot , C. Debiemme‐Chouvy , and O. Sel , “Interface Properties of 2D Graphene–Polydopamine Composite Electrodes in Protic Ionic Liquid‐Based Electrolytes Explored by Advanced Electrogravimetry,” ACS Applied Energy Materials 5 (2022): 14934–14944, 10.1021/acsaem.2c02404.

[62] N. L. Torad , S. Zhang , W. A. Amer , et al., “Advanced Nanoporous Material–Based QCM Devices: a New Horizon of Interfacial Mass Sensing Technology,” Advanced Materials Interfaces 6, no. 20 (2019): 1900849, 10.1002/admi.201900849.

[63] L. Daikhin , E. Gileadi , G. Katz , V. Tsionsky , M. Urbakh , and D. Zagidulin , “Influence of Roughness on the Admittance of the Quartz Crystal Microbalance Immersed in Liquids,” Analytical Chemistry 74 (2002): 554–561, 10.1021/ac0107610. 11838676

[64] N. Shpigel , M. D. Levi , S. Sigalov , et al., “Situ Hydrodynamic Spectroscopy for Structure Characterization of Porous Energy Storage Electrodes,” Nature Materials 15, no. 5 (2016): 570–575, 10.1038/nmat4577. 26928637

[65] M. Urbakh and L. Daikhin , “Surface Morphology and the Quartz Crystal Microbalance Response in Liquids,” Colloids and Surfaces A: Physicochemical and Engineering Aspects 134 (1998): 75–84, 10.1016/S0927-7757(97)00334-8.

[66] L. Daikhin and M. Urbakh , “Effect of Surface Film Structure on the Quartz Crystal Microbalance Response in Liquids,” Langmuir 12 (1996): 6354–6360, 10.1021/la950763d.

[67] L. Niu , L. Yang , J. Yang , et al., “Understanding the Charging of Supercapacitors by Electrochemical Quartz Crystal Microbalance,” Industrial Chemistry & Materials 1 (2023): 175–187, 10.1039/D2IM00038E.

[68] L. Daikhin , S. Sigalov , M. D. Levi , G. Salitra , and D. Aurbach , “Quartz Crystal Impedance Response of Nonhomogenous Composite Electrodes in Contact with Liquids,” Analytical Chemistry 83 (2011): 9614–9621, 10.1021/ac202410q. 22060259

[69] M. D. Levi , S. Sigalov , G. Salitra , et al., “In Situ Tracking of Ion Insertion in Iron Phosphate Olivine Electrodes via Electrochemical Quartz Crystal Admittance,” Journal of Physical Chemistry C 117, no. 3 (2013): 1247–1256, 10.1021/jp3117819.

[70] M. D. Levi , S. Sigalov , D. Aurbach , et al., “In Situ Electrochemical Quartz Crystal Admittance Methodology for Tracking Compositional and Mechanical Changes in Porous Carbon Electrodes,” Journal of Physical Chemistry C 117, no. 29 (2013): 14876–14889, 10.1021/jp403065y.

[71] Y. Ji , Z.‐W. Yin , Z. Yang , et al., “From Bulk to Interface: Electrochemical Phenomena and Mechanism Studies in Batteries via Electrochemical Quartz Crystal Microbalance,” Chemical Society Reviews 50 (2021): 10743–10763, 10.1039/d1cs00629k. 34605826

[72] C. Taviot‐Guého , P. Vialat , F. Leroux , et al., “Dynamic Characterization of Inter‐ and Intralamellar Domains of Cobalt‐Based Layered Double Hydroxides Upon Electrochemical Oxidation,” Chemistry of Materials 28, no. 21 (2016): 7793–7806, 10.1021/acs.chemmater.6b03061.

[73] A. R. Hillman , I. Efimov , and M. Skompska , “Dynamics of Regioregular Conducting Polymer Electrodes in Response to Electrochemical Stimuli,” Faraday Discussions 121 (2002): 423–439, 10.1039/b201427k. 12227583

[74] R. Etchenique and E. J. Calvo , “Electrochemical Quartz Crystal Microbalance Gravimetric and Viscoelastic Studies of Nickel Hydroxide Battery Electrodes,” Journal of the Electrochemical Society 148 (2001): A361, 10.1149/1.1357170.

[75] M. V. Voinova , M. Rodahl , M. Jonson , and B. Kasemo , “Viscoelastic Acoustic Response of Layered Polymer Films at Fluid‐Solid Interfaces: Continuum Mechanics Approach,” Physica Scripta 59 (1999): 391–396, 10.1238/Physica.Regular.059a00391.

[76] A. Arnau , “A Review of Interface Electronic Systems for AT‐Cut Quartz Crystal Microbalance Applications in Liquids,” Sensors 8 (2008): 370–411, 10.3390/s8010370. 27879713 PMC3681153

[77] M. Rodahl , F. Höök , and B. Kasemo , “QCM Operation in Liquids: an Explanation of Measured Variations in Frequency and Q Factor with Liquid Conductivity,” Analytical Chemistry 68 (1996): 2219–2227, 10.1021/ac951203m. 21619308

[78] N. Shpigel , M. D. Levi , S. Sigalov , et al., “Non‐Invasive in Situ Dynamic Monitoring of Elastic Properties of Composite Battery Electrodes by EQCM‐D,” Angewandte Chemie International Edition 54 (2015): 12353–12356, 10.1002/anie.201501787. 25916858

[79] J. Miranda , P. L. Taberna , and P. Simon , “Operando Gravimetric and Energy Loss Analysis of Na_3_V_2_(PO_4_)_2_F_3_ Composite Films by Electrochemical Quartz Microbalance with Dissipation Monitoring,” ACS Nano 19 (2025): 2419–2426, 10.1021/acsnano.4c13052. 39772442

[80] J. Surgailis , L. Q. Flagg , L. J. Richter , et al., “The Role of Side Chains and Hydration on Mixed Charge Transport in n‐Type Polymer Films,” Advanced Materials 36, no. 51 (2024): 2313121, 10.1002/adma.202313121. 38554042 PMC11656037

[81] J. Agrisuelas , C. Gabrielli , J. J. García‐Jareño , H. Perrot , O. Sel , and F. Vicente , “Viscoelastic Potential‐Induced Changes in Acoustically Thin Films Explored by Quartz Crystal Microbalance with Motional Resistance Monitoring,” Electrochimica Acta 176 (2015): 1454–1463, 10.1016/j.electacta.2015.07.131.

[82] R. A. Etchenique and E. J. Calvo , “Gravimetric Measurement in Redox Polymer Electrodes with the EQCM beyond the Sauerbrey Limit,” Electrochemistry Communications 1 (1999): 167–170, 10.1016/S1388-2481(99)00031-4.

[83] C. Leppin , A. Pomorska , M. Morga , et al., “Swelling Degree of Polyelectrolyte Layers Determined by an Electrochemical Quartz Crystal Microbalance,” Biomacromolecules 26 (2025): 914–928, 10.1021/acs.biomac.4c01205. 39838519 PMC11815823

[84] C. Yan , R. Xu , Y. Xiao , et al., “Toward Critical Electrode/Electrolyte Interfaces in Rechargeable Batteries,” Advanced Functional Materials 30, no. 23 (2020): 1909887, 10.1002/adfm.201909887.

[85] S. P. Kühn , K. Edström , M. Winter , et al., “Face to Face at the Cathode Electrolyte Interphase: from Interface Features to Interphase Formation and Dynamics,” Advanced Materials Interfaces 9, no. 8 (2022): 2102078, 10.1002/admi.202102078.

[86] J. W. Gittins , K. Ge , C. J. Balhatchet , P.‐L. Taberna , P. Simon , and A. C. Forse , “Understanding Electrolyte Ion Size Effects on the Performance of Conducting Metal–Organic Framework Supercapacitors,” Journal of the American Chemical Society 146 (2024): 12473–12484, 10.1021/jacs.4c00508. 38716517 PMC11082900

[87] Y. C. Wu , J. Ye , G. Jiang , et al., “Electrochemical Characterization of Single Layer Graphene/Electrolyte Interface: Effect of Solvent on the Interfacial Capacitance,” Angewandte Chemie International Edition 60, no. 24 (2021): 13317–13322, 10.1002/anie.202017057. 33555100 PMC8252098

[88] B. R. P. Yip , C. Chen , Y. Jiang , D. Ohayon , G. C. Bazan , and X. Wang , “Aqueous Asymmetric Pseudocapacitor Featuring High Areal Energy and Power Using Conjugated Polyelectrolytes and Ti_3_C_2_T_x_ Mxene,” Nature Communications 16 (2025): 7984, 10.1038/s41467-025-63034-9. PMC12381132 40858545

[89] S. B. Nambiar , B. Daffos , J. Segalini , P. Simon , and P.‐L. Taberna , “Probing the Role of Isoelectric Point in Charge Storage Mechanisms of Functionalized Carbon by Electrochemical Quartz Crystal Microbalance (EQCM),” Electrochemistry Communications 180 (2025): 108054, 10.1016/j.elecom.2025.108054.

[90] W. Gao , C. Debiemme‐Chouvy , M. Lahcini , H. Perrot , and O. Sel , “Tuning Charge Storage Properties of Supercapacitive Electrodes Evidenced by in Situ Gravimetric and Viscoelastic Explorations,” Analytical Chemistry 91 (2019): 2885–2893, 10.1021/acs.analchem.8b04886. 30632362

[91] L. Niu , L. Zeng , D. Yu , S. Cheng , and G. Feng , “Overscreening‐Driven Modulation of Ion Adsorption and Desorption in Conductive MOF Electrodes by Charging Rates,” ACS Nano 19 (2025): 2581–2590, 10.1021/acsnano.4c14258. 39772463

[92] Z. Yang , N. J. Leon , C. Liao , et al., “Effect of Salt Concentration on the Interfacial Solvation Structure and Early Stage of Solid‐Electrolyte Interphase Formation in Ca(BH_4_)_2_/THF for Ca Batteries,” ACS Applied Materials Interfaces 15, no. 20 (2023): 25018–25028, 10.1021/acsami.3c01606. 37171170

[93] A. Nimkar , F. Malchick , B. Gavriel , et al., “Influences of Cations' Solvation on Charge Storage Performance in Polyimide Anodes for Aqueous Multivalent Ion Batteries,” ACS Energy Letters 6 (2021): 2638–2644, 10.1021/acsenergylett.1c01007.

[94] M. Turgeman , G. Bergman , A. Nimkar , et al., “Unique Mechanisms of Ion Storage in Polyaniline Electrodes for Pseudocapacitive Energy Storage Devices Unraveled by EQCM‐D Analysis,” ACS Applied Materials & Interfaces 14 (2022): 47066–47074, 10.1021/acsami.2c13771. 36214734

[95] B. Gavriel , N. Shpigel , F. Malchik , et al., “Enhanced Performance of Ti_3_C_2_T* _x_ * (MXene) Electrodes in Concentrated ZnCl_2_ Solutions: a Combined Electrochemical and EQCM‐D Study,” Energy Storage Materials 38 (2021): 535–541, 10.1016/j.ensm.2021.03.027.

[96] J. Yun , V. Natu , I. Echols , et al., “Anion Identity and Time Scale Affect the Cation Insertion Energy Storage Mechanism in Ti_3_C_2_T_x_ Mxene Multilayers,” ACS Energy Letters 7 (2022): 1828–1834, 10.1021/acsenergylett.2c00481.

[97] Y. Zhang , S. W. Zhang , Y. Chu , et al., “Redefining Closed Pores in Carbons by Solvation Structures for Enhanced Sodium Storage,” Nature Communications 16, no. 1 (2025): 3634, 10.1038/s41467-025-59022-8. PMC12003850 40240373

[98] J. Karol , C. O. Ogolla , M. Sotoudeh , et al., “Nanoconfinement Geometry of Pillared V_2_O_5_ Determines Electrochemical Ion Intercalation Mechanisms, Storage Sites, and Diffusion Pathways,” ACS Nano 19 (2025): 26904–26919, 10.1021/acsnano.5c08169. 40659328 PMC12312152

[99] G. Yang , Q. Zhang , Z. Liu , et al., “Pore Sieving and Surficial Charge‐Driven Desolvation for High Spatial Charge Density Carbon Cathodes in Zinc‐Ion Hybrid Capacitors,” Advanced Energy Materials 15, no. 38 (2025): 2501358, 10.1002/aenm.202501358.

[100] A. K. K. Padinjareveetil and M. Pumera , “Downsizing Nanoarchitectonics of Multilayered Mxenes Electrocatalysts towards Real Time Ion Tracking via EQCM and Electrocatalytic Applications,” Carbon 226 (2024): 119228, 10.1016/j.carbon.2024.119228.

[101] J. Deng , G. Xue , C. Li , et al., “Accelerating Ion Desolvation via Bioinspired Ion Channel Design in Nonconcentrated Aqueous Electrolytes,” Journal of the American Chemical Society 147, no. 7 (2025): 5943–5954, 10.1021/jacs.4c15443. 39907055

[102] Q. Guo , S. Han , Y. Lu , et al., “Cation‐Self‐Shielding Strategy Promises High‐Voltage All‐Prussian‐Blue‐Based Aqueous K‐Ion Batteries,” Nature Communications 16, no. 1 (2025): 4707, 10.1038/s41467-025-59980-z. PMC12092807 40394062

[103] K. Mao , J. Shao , Y. Huang , et al., “Plasma‐Induced Oxygen‐Rich Vanadium Hexacyanoferrate Embedded in Hierarchical Porous Carbon Framework for Superior Aqueous Manganese‐Ion Storage,” Advanced Functional Materials 36, no. 14 (2025): 17468, 10.1002/adfm.202517468.

[104] S. Cheng , W. Gao , Z. Cao , Y. Yang , E. Xie , and J. Fu , “Selective Center Charge Density Enables Conductive 2D Metal−Organic Frameworks with Exceptionally High Pseudocapacitance and Energy Density for Energy Storage Devices,” Advanced Materials 34, no. 14 (2022): 2109870, 10.1002/adma.202109870. 35112396

[105] T. Ma , Y. Yang , D. Johnson , et al., “Understanding the Mechanism of a Conjugated Ladder Polymer as a Stable Anode for Acidic Polymer‐Air Batteries,” Joule 7 (2023): 2261–2273, 10.1016/j.joule.2023.08.009.

[106] S. Cheng , Z. Zhang , J. Yan , T. Yang , J. Zhang , and J. Fu , “Revolutionizing Micro‐Supercapacitors: Tuning MnO_2_ Electrode Polarity and Redox Activity for Superior Energy Storage,” Advanced Functional Materials 35, no. 39 (2025): 2502526, 10.1002/adfm.202502526.

[107] J. Li , K. Ge , A. O. Grammenos , et al., “Understanding Multi‐Stage Charge Storage on Nanoporous Carbons in Zn‐Ion Hybrid Capacitors,” Advanced Materials 37, no. 24 (2025): 2502422, 10.1002/adma.202502422. 40326227 PMC12177857

[108] S. Wang , K. Ji , S. Yao , et al., “Fast‐Charging Aqueous Zinc Batteries Enabled by Enhanced O–H Bond Cleavage via D‐p Spin Exchange Interactions,” ACS Energy Letters 10 (2025): 2986–2996, 10.1021/acsenergylett.5c00836.

[109] J. Zhang , W. Li , J. Wang , et al., “Engineering P‐Band Center of Oxygen Boosting H^+^ Intercalation in Δ‐MnO_2_ for Aqueous Zinc Ion Batteries,” Angewandte Chemie International Edition 62, no. 8 (2023): 202215654, 10.1002/anie.202215654. 36565058

[110] K. Hua , Q. Ma , Y. Liu , et al., “High‐Performance Bipolar Small‐Molecule Organic Cathode for Wide‐Temperature‐Range Aqueous Zinc‐Ion Batteries,” ACS Nano 19 (2025): 14249–14261, 10.1021/acsnano.5c00833. 40179152

[111] R. Wang , Y. Zhang , C. Ma , et al., “A High‐Voltage Organic Cathode Enabled by a Continuous Electronegativity Zone in Aqueous Zinc‐Organic Batteries,” Advanced Functional Materials 35, no. 38 (2025): 2505318, 10.1002/adfm.202505318.

[112] D. Wang , R. Li , J. Dong , et al., “Bidentate Coordination Enables Anions‐Regulated Solvation Structure for Advanced Aqueous Zinc Metal Batteries,” Angewandte Chemie International Edition 64, no. 2 (2025): 202414117, 10.1002/anie.202414117. 39315791

[113] C. Dong , H. Ji , T. Yang , et al., “Janus Adsorption of Polar Ion Hubs for Simultaneous Regulation of Zinc Deposition Kinetics and Interfacial Stability in Long‐Cycle Aqueous Zinc‐Ion Batteries,” Advanced Functional Materials 36, no. 2 (2026): 13529, 10.1002/adfm.202513529.

[114] Y. Pan , D. Feng , Y. Xie , Y. Jiao , and P. Wu , “Weak Dipole Effect Customized Zinc Ion‐Rich Protective Layer for Lean‐Electrolyte Zinc Metal Batteries,” Advanced Materials 37, no. 28 (2025): 2501004, 10.1002/adma.202501004. 40277190

[115] Z. Peng , S. Li , L. Tang , et al., “Water‐Shielding Electric Double Layer and Stable Interphase Engineering for Durable Aqueous Zinc‐Ion Batteries,” Nature Communications 16, no. 1 (2025): 4490, 10.1038/s41467-025-59830-y. PMC12078634 40368963

[116] Y. M. Li , W. H. Li , X. Y. Zhang , et al., “Multifunctional pH‐Controlling Electrolyte Enables Ultrastable and Highly Reversible Zinc Anode,” Advanced Functional Materials 35, no. 15 (2024): 2420446, 10.1002/adfm.202420446.

[117] S. Zheng , Y. Wang , B. Luo , et al., “Compacting Surface Charge Layers for Efficient Charge Transfer toward Stable Zn Anodes,” Energy & Environmental Science 18 (2025): 5319–5332, 10.1039/d5ee01092f.

[118] J. Bu , P. Liu , G. Ou , et al., “Interfacial Adsorption Layers Based on Amino Acid Analogues to Enable Dual Stabilization toward Long‐Life Aqueous Zinc Iodine Batteries,” Advanced Materials 37, no. 19 (2025): 2420221, 10.1002/adma.202420221. 40136081

[119] G. Duan , K. Zhang , Y. Wang , et al., “Nucleation‐Driven Volcano Effect via Interface Synergy for Stable Zn‐Ion Batteries,” Energy Storage Materials 83 (2025): 104619, 10.1016/j.ensm.2025.104619.

[120] P. Cai , X. He , K. Wang , et al., “Built‐in Electric Field Effects Tailoring Solvation Sheath and Desolvation Processes of Solvated Zn^2+^ toward Stable Aqueous Rocking‐Chair Zinc‐Ion Batteries,” Carbon Energy 7, no. 5 (2025): 691, 10.1002/cey2.691.

[121] J. Li , S. Sun , H. Huang , et al., “Ferrocyanide ”Skin“‐Mediated Anticatalysis: Mitigating Self‐Discharge in Aqueous Electrochemical Devices,” Journal of the American Chemical Society 147, no. 8 (2025): 6886–6896, 10.1021/jacs.4c16996. 39940118

[122] Y. Xu , G. Zhang , X. Wang , et al., “Efficient Modulation D/P‐Band Center Proximity in Birnessite‐Type MnO_2_ by Cation/Anion Co‐Doping for Enhanced Dual‐Ion Storage,” Advanced Functional Materials 35, no. 27 (2025): 2500137, 10.1002/adfm.202500137.

[123] M. Xue , M. Cao , C. Xu , D. Xiao , and X. Zhang , “EQCM Investigation of a Dual‐Doped Polymer Electrode for Li‐Ion Batteries with Improved Reversible Capacity,” ACS Applied Materials & Interfaces 14 (2022): 25584–25591, 10.1021/acsami.2c06004. 35622015

[124] Z. Li , Y. Liu , S. Yang , et al., “Probing Capacity Decay in Vanadium Oxide Cathodes of Aqueous Zinc‐Ion Batteries Using Operando EQCM‐D,” ACS Energy Letters 10 (2025): 2821–2830, 10.1021/acsenergylett.5c00901.

[125] X.‐L. Li , H. Song , Y.‐H. Zhang , et al., “Capacitance Decay Mechanism of Vanadium Nitride Supercapacitor Electrodes in KOH Electrolytes,” Rare Metals 44, no. 6 (2025): 3909–3919, 10.1007/s12598-025-03245-7.

[126] Q. Sun , H. Liu , Y. Fan , et al., “Aging‐Induced Specific Adsorption of Cations Thickening Electric Double Layer in Supercapacitors at 3.0 V,” Journal of Power Sources 651 (2025): 237548, 10.1016/j.jpowsour.2025.237548.

[127] J. Huang , L. Xue , Y. Huang , et al., “Thermodynamically Spontaneously Intercalated H_3_O^+^ Enables LiMn_2_O_4_ with Enhanced Proton Tolerance in Aqueous Batteries,” Nature Communications 15, no. 1 (2024): 6666, 10.1038/s41467-024-51060-y. PMC11303759 39107315

[128] T. Ma , C.‐H. Li , R. M. Thakur , D. P. Tabor , and J. L. Lutkenhaus , “The Role of the Electrolyte in Non‐Conjugated Radical Polymers for Metal‐Free Aqueous Energy Storage Electrodes,” Nature Materials 22 (2023): 495–502, 10.1038/s41563-023-01518-z. 36973544

[129] L. Wu , Z. Li , Y. Xiang , et al., “Unraveling the Charge Storage Mechanism of β‐MnO_2_ in Aqueous Zinc Electrolytes,” ACS Energy Letters 9 (2024): 5801–5809, 10.1021/acsenergylett.4c02559.

[130] A. K. K. Padinjareveetil , M. Pykal , A. Bakandritsos , R. Zboril , M. Otyepka , and M. Pumera , “Real Time Tracking of Nanoconfined Water‐Assisted Ion Transfer in Functionalized Graphene Derivatives Supercapacitor Electrodes,” Advanced Science 11, no. 39 (2024): 2307583, 10.1002/advs.202307583. 39107963 PMC11497090

[131] L. Li , W. Liu , H. Dong , et al., “Surface and Interface Engineering of Nanoarrays toward Advanced Electrodes and Electrochemical Energy Storage Devices,” Advanced Materials 33, no. 13 (2021): 202004959, 10.1002/adma.202004959. 33615578

[132] L. He , S. Wang , F. Yu , and Y. Chen , “Sluggish Li_2_O_2_ Dissolution—A Key to Unlock High‐Capacity Lithium–Oxygen Batteries,” Chemical Science 16 (2025): 627–636, 10.1039/d4sc05911e. 39650218 PMC11621826

[133] K. Hu , T. Hu , T. Yao , et al., “Photoassisted Li‐Co_2_ Batteries with Ultrahigh Energy Efficiency and Cycle Stability by a Redox Mediator,” ACS Nano 19, no. 8 (2025): 7707–7717, 10.1021/acsnano.4c11559. 39977540

[134] K. Yang , Y. Li , L. Jia , et al., “Atomic/Nano‐Scale in‐Situ Probing the Shuttling Effect of Redox Mediator in Na–O_2_ Batteries,” Journal of Energy Chemistry 56 (2021): 438–443, 10.1016/j.jechem.2020.08.025.

[135] B. W. Schick , V. Vanoppen , M. Uhl , et al., “Influence of Chloride and Electrolyte Stability on Passivation Layer Evolution at the Negative Electrode of Mg Batteries Revealed by Operando EQCM‐D,” Angewandte Chemie International Edition 63, no. 52 (2024): 202413058, 10.1002/anie.202413058. PMC11656148 39523208

[136] W. Zhang , Y. Liu , X. Luo , et al., “Multi‐Solvent Synergy Strategy Unlocks Anti‐Corrosion and High Reversibility of Zinc Anodes: Paving the Way for Robust and Temperature‐Resilient Zinc‐Iodine Batteries,” Advanced Functional Materials 35, no. 51 (2025): 12633, 10.1002/adfm.202512633.

[137] W. Zhang , Q. Zhao , Y. Hou , et al., “Dynamic Interphase–Mediated Assembly for Deep Cycling Metal Batteries,” Science Advances 7, no. 49 (2021): abl3752, 10.1126/sciadv.abl3752. PMC8635427 34851670

[138] X.‐B. Cheng , C. Yan , X.‐Q. Zhang , H. Liu , and Q. Zhang , “Electronic and Ionic Channels in Working Interfaces of Lithium Metal Anodes,” ACS Energy Letters 3 (2018): 1564–1570, 10.1021/acsenergylett.8b00526.

[139] J. Xiao , N. Adelstein , Y. Bi , et al., “Assessing Cathode–Electrolyte Interphases in Batteries,” Nature Energy 9 (2024): 1463–1473, 10.1038/s41560-024-01639-y.

[140] C. Wang , K. Wan , P. Liu , et al., “Localized High‐Concentration Electrolytes with Semi‐Solvated Hexafluoroisopropyl Methyl Ether Diluent for Wide‐Temperature‐Range Lithium Metal Batteries,” Angewandte Chemie International Edition 64, no. 27 (2025): 202506083, 10.1002/anie.202506083. 40266257

[141] P. G. Kitz , M. J. Lacey , P. Novák , et al., “Operando EQCM‐D with Simultaneous in Situ EIS: New Insights into Interphase Formation in Li Ion Batteries,” Analytical Chemistry 91, no. 3 (2018): 2296–2303, 10.1021/acs.analchem.8b04924. 30569698

[142] P. Sayavong , W. Zhang , S. T. Oyakhire , et al., “Dissolution of the Solid Electrolyte Interphase and Its Effects on Lithium Metal Anode Cyclability,” Journal of the American Chemical Society 145 (2023): 12342–12350, 10.1021/jacs.3c03195. 37220230

[143] C. N. Hong , M. Yan , O. Borodin , et al., “Robust Battery Interphases from Dilute Fluorinated Cations,” Energy & Environmental Science 17 (2024): 4137–4146, 10.1039/d4ee00296b. 38899028 PMC11185048

[144] P. G. Kitz , P. Novák , and E. J. Berg , “Influence of Water Contamination on the SEI Formation in Li‐Ion Cells: an Operando EQCM‐D Study,” ACS Applied Materials & Interfaces 12 (2020): 15934–15942, 10.1021/acsami.0c01642. 32141729

[145] Y. Ji , Y. Huang , Z. Dong , et al., “Anion Adsorption at the Inner‐Helmholtz Plane Directs Cathode Electrolyte Interphase Formation,” Angewandte Chemie International Edition 64, no. 20 (2025): 202425535, 10.1002/anie.202425535. 40011214

[146] S. Guo , W. Li , X. Wu , et al., “Functional Separator Induced Interface Potential Uniform Reformation Enabling Dendrite‐Free Metal Batteries,” Advanced Functional Materials 35, no. 37 (2025): 2504599, 10.1002/adfm.202504599.

[147] E. Bendadesse , P. Lemaire , P. Travers , J.‐M. Tarascon , and O. Sel , “Accurate Internal Monitoring of Batteries by Embedded Piezoelectric Sensors,” Small Methods 8, no. 12 (2024): 2400472, 10.1002/smtd.202400472. 38856032

[148] W. Li , Y. Duan , S. Ge , et al., “Locking‐Chain Electrolyte Additive Enabling Moisture‐Tolerant Electrolytes for Sodium‐Ion Batteries,” Nature Communications 16, no. 1 (2025): 6405, 10.1038/s41467-025-61603-6. PMC12254247 40645968

[149] Z. Zhang , S. Said , A. J. Lovett , et al., “The Influence of Cathode Degradation Products on the Anode Interface in Lithium‐Ion Batteries,” ACS Nano 18, no. 13 (2024): 9389–9402, 10.1021/acsnano.3c10208. 38507591 PMC10993644

[150] M. R. Krumov , S. Lang , L. Johnson , and H. D. Abruña , “Operando Investigation of Solid Electrolyte Interphase Formation, Dynamic Evolution, and Degradation during Lithium Plating/Stripping,” ACS Applied Materials & Interfaces 15 (2023): 47692–47703, 10.1021/acsami.3c08485. 37751476

[151] S. Cora , S. Ahmad , and N. Sa , “Situ Probing of Mass Exchange at the Solid Electrolyte Interphase in Aqueous and Nonaqueous Zn Electrolytes with EQCM‐D,” ACS Applied Materials Interfaces 13, no. 8 (2021): 10131–10140, 10.1021/acsami.1c00565. 33596040

[152] S. Boyd , K. Ganeshan , W.‐Y. Tsai , et al., “Effects of Interlayer Confinement and Hydration on Capacitive Charge Storage in Birnessite,” Nature Materials 20 (2021): 1689–1694, 10.1038/s41563-021-01066-4. 34341525

[153] L. Liu , E. Raymundo‐Pinero , S. Sunny , et al., “Role of Surface Terminations for Charge Storage of Ti_3_C_2_T_x_ Mxene Electrodes in Aqueous Acidic Electrolyte,” Angewandte Chemie International Edition 63, no. 14 (2024): 202319238, 10.1002/anie.202319238. 38324461

[154] M. Elmanzalawy , H. Song , M. Tobis , et al., “Nanoconfinement‐Induced Electrochemical Ion‐Solvent Cointercalation in Pillared Titanate Host Materials,” Angewandte Chemie International Edition 64, no. 20 (2025): 202423593, 10.1002/anie.202423593. PMC12070453 40008459

[155] K. Xiao , X. Jiang , S. Zeng , et al., “Porous Structure‐Electrochemical Performance Relationship of Carbonaceous Electrode‐Based Zinc Ion Capacitors,” Advanced Functional Materials 34, no. 44 (2024): 2405830, 10.1002/adfm.202405830.

[156] C. Chen , C. Yang , X. Fu , et al., “Non‐Porous Two‐Dimensional Conducting Metal–Organic Frameworks with Enhanced Capacitance,” Journal of Materials Chemistry A 12 (2024): 29606–29614, 10.1039/d4ta05484a.

[157] M. D. P. Bernicola , M. Lounasvuori , J. Padilla‐Pantoja , et al., “On the Electrochemical Activation of Nanoporous Reduced Graphene Oxide Electrodes Studied by in Situ/Operando Electrochemical Techniques,” Advanced Functional Materials 34, no. 46 (2024): 2408441, 10.1002/adfm.202408441.

[158] F. W. Richey , B. Dyatkin , Y. Gogotsi , and Y. A. Elabd , “Ion Dynamics in Porous Carbon Electrodes in Supercapacitors Using in Situ Infrared Spectroelectrochemistry,” Journal of the American Chemical Society 135 (2013): 12818–12826, 10.1021/ja406120e. 23915377

[159] A. C. Forse , J. M. Griffin , C. Merlet , et al., “NMR Study of Ion Dynamics and Charge Storage in Ionic Liquid Supercapacitors,” Journal of the American Chemical Society 137 (2015): 7231–7242, 10.1021/jacs.5b03958. 25973552 PMC4500645

[160] J. Yuan , T. Sun , J. Chen , et al., “Microbial Surface Confined Growth Strategy for the Synthesis of Highly Loaded NiCoP Nanoparticles with Hollow Derived Carbon Shells for Sodium Ion Capture,” Advanced Science 12, no. 1 (2024): 2407616, 10.1002/advs.202407616. 39465908 PMC11714236

[161] J. T. Mefford , A. R. Akbashev , M. Kang , et al., “Correlative Operando Microscopy of Oxygen Evolution Electrocatalysts,” Nature 593 (2021): 67–73, 10.1038/s41586-021-03454-x. 33953412

[162] G. Yang , Y. Yang , Y. Li , F. Song , and Q. Chen , “A Double‐Confined Strategy for Enhancing the Pseudocapacitance Performance of Nickel‐Based Sulfides‐Unveiling Aqueous Pseudocapacitive Energy Storage Mechanism,” Journal of Colloid and Interface Science 686 (2025): 1089–1104, 10.1016/j.jcis.2025.02.027. 39933347

[163] M. E. Carbone , R. Ciriello , S. Granafei , A. Guerrieri , and A. M. Salvi , “EQCM and XPS Investigations on the Redox Switching of Conducting Poly(O‐Aminophenol) Films Electrosynthesized onto Pt Substrates,” Electrochimica Acta 176 (2015): 926–940, 10.1016/j.electacta.2015.07.047.

[164] C. Malacrida , K. Dirnberger , E. M. Halim , O. Sel , H. Perrot , and S. Ludwigs , “Ion Charge Compensation Upon Electrochemical Doping of Redox Polymer Films with Tunable Crosslinking Density,” Advanced Electronic Materials 12, no. 6 (2026): 00645, 10.1002/aelm.202500645.

[165] M. Stich , C. Leppin , F. T. Krauss , et al., “Comparing the SEI Formation on Copper and Amorphous Carbon: a Study with Combined Operando Methods,” Batteries 11 (2025): 273, 10.3390/batteries11070273.

[166] E. Wang , S. Ge , W. Li , et al., “Precisely Deciphering Solid Electrolyte Interphase,” Matter 8, no. 11 (2025): 102368, 10.1016/j.matt.2025.102368.

[167] A. Lahiri , G. Pulletikurthi , N. Behrens , et al., “Situ Atomic Force Microscopy and Electrochemical Quartz Crystal Microbalance Studies on the Electrodeposition and Oxidation of Silicon,” Journal of Physical Chemistry C 122, no. 26 (2018): 14499–14510, 10.1021/acs.jpcc.8b02462.

[168] T. Carstens , A. Ispas , N. Borisenko , et al., “In Situ Scanning Tunneling Microscopy (STM), Atomic Force Microscopy (AFM) and Quartz Crystal Microbalance (EQCM) Studies of the Electrochemical Deposition of Tantalum in Two Different Ionic Liquids with the 1‐Butyl‐1‐Methylpyrrolidinium Cation,” Electrochimica Acta 197 (2016): 374–387, 10.1016/j.electacta.2015.07.178.

[169] J. M. Griffin , A. C. Forse , W.‐Y. Tsai , et al., “In Situ NMR and Electrochemical Quartz Crystal Microbalance Techniques Reveal the Structure of the Electrical Double Layer in Supercapacitors,” Nature Materials 14, no. 8 (2015): 812–819, 10.1038/nmat4318. 26099110

[170] E. Zhang , Y.‐C. Wu , H. Shao , et al., “Unraveling the Capacitive Charge Storage Mechanism of Nitrogen‐Doped Porous Carbons by EQCM and ssNMR,” Journal of the American Chemical Society 144, no. 31 (2022): 14217–14225, 10.1021/jacs.2c04841. 35914237

[171] C. J. Balhatchet , J. W. Gittins , S.‐J. Shin , et al., “Revealing Ion Adsorption and Charging Mechanisms in Layered Metal–Organic Framework Supercapacitors with Solid‐State Nuclear Magnetic Resonance,” Journal of the American Chemical Society 146 (2024): 23171–23181, 10.1021/jacs.4c05330. 39133641 PMC11345813

[172] X. Huang , Y. F. Cheng , H. Liu , et al., “Interlayer Confined Capacitive Response via Solvated Cointercalation in Graphite Layers,” ACS Nano 19, no. 7 (2025): 7168–7177, 10.1021/acsnano.4c16593. 39933132

[173] J. Miranda , G. Franklin , T. S. Mathis , P.‐L. Taberna , and P. Simon , “Unraveling the Two‐Phase Lithiation Process in TiS_2_ by Using the Combination of Operando EQCM and Electrochemical Dilatometry Techniques,” Energy Storage Materials 65 (2024): 103105, 10.1016/j.ensm.2023.103105.

[174] L. Liu , Y.‐C. Wu , L. Huang , et al., “Alkali Ions Pre‐Intercalated Layered MnO_2_ Nanosheet for Zinc‐Ions Storage,” Advanced Energy Materials 11, no. 31 (2021): 2101287, 10.1002/aenm.202101287.

[175] K. Ge , H. Shao , E. Raymundo‐Piñero , P.‐L. Taberna , and P. Simon , “Cation Desolvation‐Induced Capacitance Enhancement in Reduced Graphene Oxide (RGO),” Nature Communications 15 (2024): 1935, 10.1038/s41467-024-46280-1. PMC10908864 38431624

[176] K. Betts , Y. Jiang , M. Frailey , K. Yohannes , and Z. Feng , “Potential‐Dependent ATR‐Seiras and EQCM‐D Analysis of Interphase Formation in Zinc Battery Electrolytes,” ACS Applied Materials & Interfaces 16 (2024): 63026–63038, 10.1021/acsami.4c15318. 39492667

[177] E. K. Ulker , P. Hirani , S. Guan , and A. Lahiri , “Combined in Situ EQCM‐Raman Study of Zn Storage Mechanism in Polyaniline for Zinc‐Ion Battery,” Small Methods 9, no. 11 (2025): 01273, 10.1002/smtd.202501273. PMC12641370 41030216

[178] R. Wang , Q. Ma , L. Zhang , et al., “An Aqueous Electrolyte Regulator for Highly Stable Zinc Anode under ‐35 to 65°C,” Advanced Energy Materials 13, no. 40 (2023): 2302543, 10.1002/aenm.202302543.

[179] A. Perju , D. Zhang , R. J. Wang , P.‐L. Taberna , Y. Gogotsi , and P. Simon , “Operando Tracking of Resistance, Thickness, and Mass of Ti_3_C_2_T* _X_ * MXene in Water‐in‐Salt Electrolyte,” Advanced Energy Materials 15, no. 20 (2025): 2405028, 10.1002/aenm.202405028.

[180] A. Duan , S. Luo , Y. Tang , et al., “Situ Monitoring of Dynamic Adsorption‐Induced Interfacial Buffering toward Highly Stable Zinc Metal Batteries,” Advanced Energy Materials 15, no. 19 (2025): 2404693, 10.1002/aenm.202404693.

[181] E. Bendadesse , C. Gervillie‐Mouravieff , C. Leau , et al., “Spotting Interface Structuring during Na‐Insertion into the NaSICON Na_3_V_2_(PO_4_)_3_ by EQCM and Operando Fiber Optic Infrared Spectroscopy,” Advanced Energy Materials 13, no. 26 (2023): 2300930, 10.1002/aenm.202300930.

